# Exosomal circZNF451 restrains anti-PD1 treatment in lung adenocarcinoma via polarizing macrophages by complexing with TRIM56 and FXR1

**DOI:** 10.1186/s13046-022-02505-z

**Published:** 2022-10-08

**Authors:** Jian Gao, Yong-Qiang Ao, Ling-Xian Zhang, Jie Deng, Shuai Wang, Hai-Kun Wang, Jia-Hao Jiang, Jian-Yong Ding

**Affiliations:** 1 Department of Thoracic Surgery, Zhongshan Hospital, Fudan University, 180 Fenglin Road, Shanghai, 200032 People’s Republic of China; 2 Cancer Center, Zhongshan Hospital, Fudan University, Shanghai, China; 3 Department of Cardiothoracic Surgery, The Second Affiliated Hospital of Nanchang University, Nanchang, Jiangxi China; 4 Institute of Vascular Disease, Shanghai TCM-Integrated Hospital, Shanghai University of Traditional Chinese Medicine, Shanghai, China; 5 CAS Key Laboratory of Molecular Virology and Immunology, Institute Pasteur of Shanghai, Chinese Academy of Sciences, Shanghai, China

**Keywords:** Lung adenocarcinoma, PD1 blockade resistance, Exosomal circRNA, Immunotherapy

## Abstract

**Background:**

Although success was achieved in the therapy for a minority of advanced lung adenocarcinoma (LUAD) patients, anti-programmed death 1 (PD1) resistance was found in most LUAD patients. Here, we aimed to uncover a potential role of exosomal circular RNAs (circRNAs) in LUAD refractory to PD1 blockade.

**Methods:**

circRNA sequencing and qRT-PCR were performed to determine the level of exosomal circRNAs in LUAD patients subsequently treated with anti-PD1. Then, the RNA pulldown, RNA immunoprecipitation, mass spectrometry, chromatin immunoprecipitation, luciferase reporter assays, flow cytometry, RNA sequencing, and in vitro and in vivo models were used to uncover the biological functions and underlying mechanism of circZNF451 in LUAD anti-PD1 treatment resistance.

**Results:**

circRNA sequencing and qRT-PCR identified the up-regulation of exosomal circZNF451 from LUAD patients with progressive disease (PD) compared to those with partial remission (PR) after PD1 blockade therapy. Furthermore, elevated circZNF451 was revealed to be associated with poor prognosis of LUAD patients. Additionally, exosomal circZNF451 was demonstrated to induce an anti-inflammatory phenotype in macrophages and exhaustion of cytotoxic CD8^+^ T cells, and enhanced TRIM56-mediated degradation of FXR1 to activate the ELF4–IRF4 pathway in macrophages. By transgenic mice, knockout of ELF4 in macrophages was found to rescue immunotherapy efficacy in tumors with high level of exosomal circZNF451.

**Conclusion:**

Exosomal circZNF451 reshapes the tumor immune microenvironment by inducing macrophages polarization via the FXR1- ELF4–IRF4 axis and is a novel biomarker for predicting the sensitivity of PD1 blockade in LUAD.

**Supplementary Information:**

The online version contains supplementary material available at 10.1186/s13046-022-02505-z.

## Background

Lung cancer has the highest cancer incidence and mortality worldwide, with a 5-year survival rate of only ~ 10–20% [1]. Within non-small cell lung cancer, lung adenocarcinoma (LUAD) is the most common histological subtype, accounting for ~ 40% of cases [2]. While radical resection with lobectomy or segmentectomy is effective in early-stage LUAD, prognosis in the advanced stage is poor [3]. Radiation and chemotherapy remain first-line therapies, and progress in the development of targeted therapies against oncogenic drivers, such as *EGFR* and *KRAS,* and recently immunotherapy targeting immune checkpoints shows promise for a new era in treating advanced LUAD [4]. Nonetheless, intrinsic and acquired resistance to immunotherapy presents a huge barrier for advanced LUAD treatment [5]. Thus, in-depth studies characterizing mechanisms that lead to immune resistance are pivotal for developing new therapeutic strategies for advanced LUAD.

Circular RNAs (circRNAs; endogenous noncoding RNAs) are closed-loop structures [6] with critical roles in the initiation and progression of cancers [7]. Their relatively stable structure, species conservation, and high abundance suggest that circRNAs may serve as reliable biomarkers for cancers [8]. CircRNAs could exist in the nucleus or cytoplasm and previous studies discovered some circRNAs enriched in exosomes [9], which are extracellular vesicles ~ 30–150 nm in diameter that facilitate communications between cells. Following discharge into extracellular fluids, exosomes are transferred to recipient cells and regulate the biological functions of other cells via content transfer. circRNAs from tumor-derived exosomes establish immune suppression and escape, and dysregulation of exosomal circRNAs in the tumor microenvironment (TME) can induce the exhaustion and dysfunction of immune cells, such as CD8^+^ T cells, natural killer (NK) cells, and dendritic cells, and elicit upregulation of immune checkpoints, such as programmed death 1 (PD1) in T cells [10]. Thus, further investigation into the relationship between exosomal circRNAs and the TME may provide a better understanding of intrinsic and acquired resistance to immunotherapy.

Immunotherapy elicits mainly cytotoxic activities in the TME, especially tumor-infiltrating cytotoxic T lymphocytes, and dysregulated immune checkpoints (e.g., PD1, PD-L1, CTLA4, TIM3) enable immune invasion of tumor cells. Several drugs that block these immune checkpoints are approved to treat various cancers in the advanced stage [11]; yet, failure of these blockades remains inevitable. This failure stems from tumor cell-intrinsic resistance determined by genetic or transcriptional profiles and dysfunction of cytotoxic T lymphocytes (especially cytotoxic CD8^+^ T cells). These processes are mediated either by direct communication between tumor cells and cytotoxic T lymphocytes or indirectly by inhibition from other subgroups in the TME (e.g., myeloid-derived suppressor cells and regulatory T [Treg] cells) [12]. Interactions between tumor cells and immune-suppressed subgroups leads to the exhaustion of antitumor immunity and orchestrated resistance to immunotherapy. Thus, a deeper understanding of communication between tumor cells and immune-suppressed tumor cells may facilitate immunotherapy progress in cancer.

Here, circRNA sequencing and qRT-PCR showed the up-regulation of circZNF451 (hsa_circ_0002638) in exosomes from patients with progressive disease (PD) compared with patients with partial remission (PR). Level of circZNF451 in exosomes was highly consistent with the expression of circZNF451 in tumor tissues. Moreover, in vitro and in vivo assays suggested that exosomal circZNF451 could induce M2 polarization of macrophages to reshape the TME and limit the sensitivity of anti-PD1 treatment. Moreover, we demonstrated that circZNF451 elicited ubiquitination of the RBP FXR1 by the E3 ligase TRIM56 to activate the ELF4-IRF4 pathway in macrophages. Importantly, conditional knockout of ELF4 in macrophages was found to enhance the efficacy of anti-PD1 in LUAD with high expression of circZNF451 by transgenic mice. Collectively, we reveal a new mechanism for the anti-PD1 resistance, and a potential biomarker for the prediction of anti-PD1 efficacy.

## Methods

### Cell culture and reagents

Six LUAD cell lines A549, H1299, Calu-3, H1975, H1395, and LLC, HEK-293 T cells, and THP-1 cells were provided by the cell bank of the Chinese Academy of Sciences. Cells were cultured with DMEM or RPMI-1640 (Gibco, USA) with 10% fetal bovine serum (Gibco, USA) at 37℃ and 5% CO_2_.

### Vector construction and plasmid transfection

All plasmids used in this study were constructed by Genomeditech (Shanghai, China). Lentivirus production and infection were performed using a lentiviral packaging kit and liposomal transfection reagent (Yeasen, Shanghai). Sequences of shcircZNF451, siFXR1, siTRIM56, siELF4, and siIRF4 are listed in Supplementary Table 3.

### Tissues samples

ALL tumor tissues and peripheral blood were obtained from patients at Fudan University Zhongshan Hospital who underwent surgery between 2008 and 2010. The tumor tissues were frozen by liquid nitrogen and stored under -80 ℃. The sera of the peripheral blood were stored under -20 ℃. The tissue microarray (TMA) consisted of 113 LUAD tissues and peripheral tumor tissues. Histopathology was evaluated by two experienced pathologists. Patient clinical characteristics are described in Supplementary Table 2. All patients signed the consent form, and all human and animal work was approved by the Ethics Committee of Fudan University Zhongshan Hospital.

### Exosome isolation

Exosomes from the sera of LUAD patients and culture medium of LUAD cell lines were extracted with ExoQuick exosome precipitation solution (SBI, USA) according to the manufacturer’s protocol. Isolated exosomes were further used for quantitative real-time PCR (qRT-PCR) and circRNA sequencing.

### Western blotting, qRT-PCR, and immunohistochemistry (IHC)

Western blotting, qRT-PCR, and IHC were performed according to our previous study [13], and details are provided in the Supplementary Methods. The antibodies used in this study were presented in supplementary Table 4. The primers for qRT-PCR were presented in supplementary Table 5. The original images of western blotting were presented in additional file 4.

### RNA sequencing and Sanger sequencing

RNA sequencing and bioinformatic analysis were performed by Majorbio (Shanghai, China). Detected circRNAs and mRNAs with fold change (FC) values of > 2 and *P* values of < 0.05 were included for further analysis. Heat map and volcano plots were used to illustrate the expression profiles of different circRNAs and mRNAs. Kyoto Encyclopedia of Genes and Genomes (KEGG) and Gene ontology (GO) analyses were used to identify immune-related genes and explore potential candidates for FXR1. Sanger sequencing of circZNF451 in A549 cells was performed by Tsingke (Shanghai, China).

### RNase R and RNase a digestion

The RNase R was used to digest the linear mRNA, while the circRNAs could endure its digestion. The RNase A could be used to digest the circRNAs. A total of 2 μg of RNA was incubated with 10 U of RNase R or RNase A (Thermo Fisher, USA) for approximately 30 min. Digested RNA was further purified with a TRIeasy Plus total RNA kit (Yeasen, Shanghai) and analyzed by qRT-PCR.

### Fluorescence in situ hybridization (FISH) immunofluorescence

To determine the localization of circZNF451, FXR1, and TRIM56 in THP-1 cells, cells were fixed with 4% formaldehyde, prehybridized, and further hybridized with fluorescein-labeled circZNF451 probe for 12 h at 37℃. Cells were blocked with goat serum for 30 min and incubated with primary antibody for 1 h. After washing with phosphate-buffered saline (PBS), cells were incubated with Alexa Fluor 488-conjugated secondary antibody (Yeasen, Shanghai) for 1 h. Cells were sealed with mounting medium plus DAPI, and images were captured with a fluorescence microscope. The probe of circZNF451 was listed in supplementary Table 5. To evaluate the expression of circZNF451 in the LUAD and peri-tumor tissues of TMA stained with FISH, we selected four represented areas in each dot of the TMA. The mean optical intensity of each area was calculated by the Image-Pro Plus software (Media Cybernetics; Silver Spring, MD, USA) and the average of the mean optical intensity in the four areas was regarded as the score for the expression of circZNF451.

### Cytokine measurement

Cytokine production in THP-1 cell supernatants was measured using the human cytokine/chemokine microarray (Luminex, USA). After the stimulation by LPS for 24 h, concentrations of interleukin-1β (IL-1β), IL-1 receptor antagonist (IL-1Ra), granulocyte–macrophage colony-stimulating factor (GM-CSF), CXCL-1, and IL-10 in the supernatant were measured by enzyme-linked immunosorbent assay (ELISA). ELISA kits used are listed in Supplementary Table 4.

### Flow cytometry analysis

To analyze immune cell infiltration in subcutaneous tumors, tumor tissues were ground into single cells, filtered with a 70-μm cell strainer, and purified by Percoll gradient (Yeasen, Shanghai). Then, ~ 2 × 10^6^ cells were washed with PBS and stained with antibodies and Fc blocker (BioLegend) for 30 min on ice. For Foxp3 staining in Treg cells, cells were permeabilized with 0.1% Triton X-100 and stained with antibody for 90 min. To stain cytokines in the cytoplasm, cells were stimulated with phorbol myristate acetate (PMA; 10 ng/mL) and ionomycin (1 μg/mL) and blocked by brefeldin A (1 μg/mL) for 4 h and stained with surface marker antibodies for 30 min. Cells were fixed, incubated with 0.2% saponin buffer for 20 min, and stained with cytokine antibodies for 30 min. Samples were analyzed with a Fortessa flow cytometer (BD), and data were analyzed with FlowJo V10.6. Antibodies used for flow cytometry are listed in Supplementary Table 4.

### Coculture assay and CD8^+^ T cell stimulation

A total of 1 × 10^6^ macrophages cells (stimulated by 100 μg/mL PMA for 24 h in THP-1 cells) were seeded in the lower chamber of six-well Transwell chambers (Corning, USA), and 5 × 10^5^ LUAD cells were seeded in the upper chamber. After 48 h, the supernatant and macrophages were collected for further analysis. After isolation of CD8^+^T cells from healthy donors by using anti-CD8 immunomagnetic beads, cells were stimulated with anti-CD3 (10 μg/mL), anti-CD28 (1.5 μg/mL), and IL-2 (200 U/mL) and cultured with supernatant from the coculture system for 48 h. The proliferation of CD8^+^ T cells was detected by CFSE according to the manufacturer’s protocol (biolegend, USA) and analyzed by the flow cytometry.

### Coimmunoprecipitation (Co-IP)

To evaluate interactions between FXR1 and TRIM56, THP-1 cells were lysed with co-IP buffer supplemented with proteinase, RNase, and phosphatase inhibitors and centrifuged at 12,000 rpm for 15 min. The supernatant was incubated with the beads, which were incubated with FXR1 antibody. After 12 h, beads were washed with lysis buffer, mixed with sodium dodecyl sulfate (SDS) loading buffer, and analyzed by Western blotting.

### RNA pulldown

The RNA pulldown assay was performed with an RNA antisense purification kit from BersinBio (Guangzhou, China). Briefly, a total of 2 × 10^7^ cells were lysed with lysis buffer supplemented with proteinase and RNase inhibitor and centrifuged at 16,000 × *g* for 10 min. The supernatant was incubated with 40 pmol of biotinylated circZNF451 probe for 3 h at 37℃. Twenty microliters of streptavidin beads were washed and hybridized with the supernatant for 30 min at room temperature. Enriched proteins bound to beads were eluted and used for mass spectrometry and western blotting.

### RNA immunoprecipitation (RIP)

RIP was performed using a Magna RIP kit (Millipore, USA) according to the manufacturer’s protocol. A total of 10 [7] cells were fixed, lysed, and centrifuged, and the supernatant was hybridized with the streptavidin beads for 12 h. RNA was eluted from the beads, and qRT-PCR was performed.

### Chromatin immunoprecipitation (ChIP)

Cells (6 × 10^6^) were cross-linked with 1% formaldehyde for 10 min and quenched with 0.2 g of glycine. ChIP was performed with a SimpleChIP Plus sonication chromatin IP kit (Cell Signaling Technology, USA) according to the manufacturer’s protocol. The mixture was digested, and DNA was sonicated into 200- to 800-base pair (bp) fragments. After incubation with ELF4 antibody (Santa Cruz, USA) for 12 h, the DNA–protein complexes were purified with magnetic beads, and eluted DNA was analyzed by qRT-PCR.

### Luciferase reporter assay

Wild-type (WT) and mutant IRF4 promoters were copied into pEZX-FR01 and cotransfected into HEK293T cells with the ELF4 overexpression plasmid. After 48 h, cells were lysed, and a luciferase reporter assay kit (Promega, USA) was used to measure Renilla and firefly luciferase activity.

### In vivo experiments

C57BL/6 J mice were obtained from the Vital River (Beijing, China). C57BL/6-Lyz2^CreERT2^ and C57BL/6 J-ELF4^em1(flox)Smoc^ mice were constructed by the Shanghai Model Organisms Center (Shanghai, China). A total of forty-five mice were used in this study and each group contained five ones. The mice were fed in a pathogen-free environment. Before the subcutaneous implantation of LLC cells or sacrifice, the mice were anesthetized by 1% Pentobarbital sodium (80 mg/kg). Mice with conditional knockout of ELF4 in macrophages were constructed by C57BL/6-Lyz2^CreERT2^ × C57BL/6 J-ELF4^em1(flox)Smoc^ cross. ELF4 knockout in macrophages was performed by tamoxifen administration (2 mg/mouse) for 5 days. The subcutaneous xenograft model is detailed in the Supplementary Methods.

### Statistical methods

SPSS 23.0 (IBM, USA) and GraphPad Prism 8.0 were used to analyze statistical differences. The clinical characteristics were analyzed by chi-squared tests. Differences between two groups were analyzed by Student’s *t* test, and Spearman correlation analysis was performed to determine correlation coefficients. For three groups or more, the one-way ANOVA test was used to compare the difference between groups. Univariate and multivariate Cox regression analysis was applied to find the independent risk factors for LUAD prognosis. Kaplan–Meier and log-rank tests were used to compare survival in different groups. A *P* value of < 0.05 was regarded as statistically significant; *P < 0.05; **P < 0.01; ***P < 0.001.

## Results

### circZNF451 in the prognosis of LUAD patients and the anti-PD1 therapy of advanced LUAD patients

**Fig. 1:**
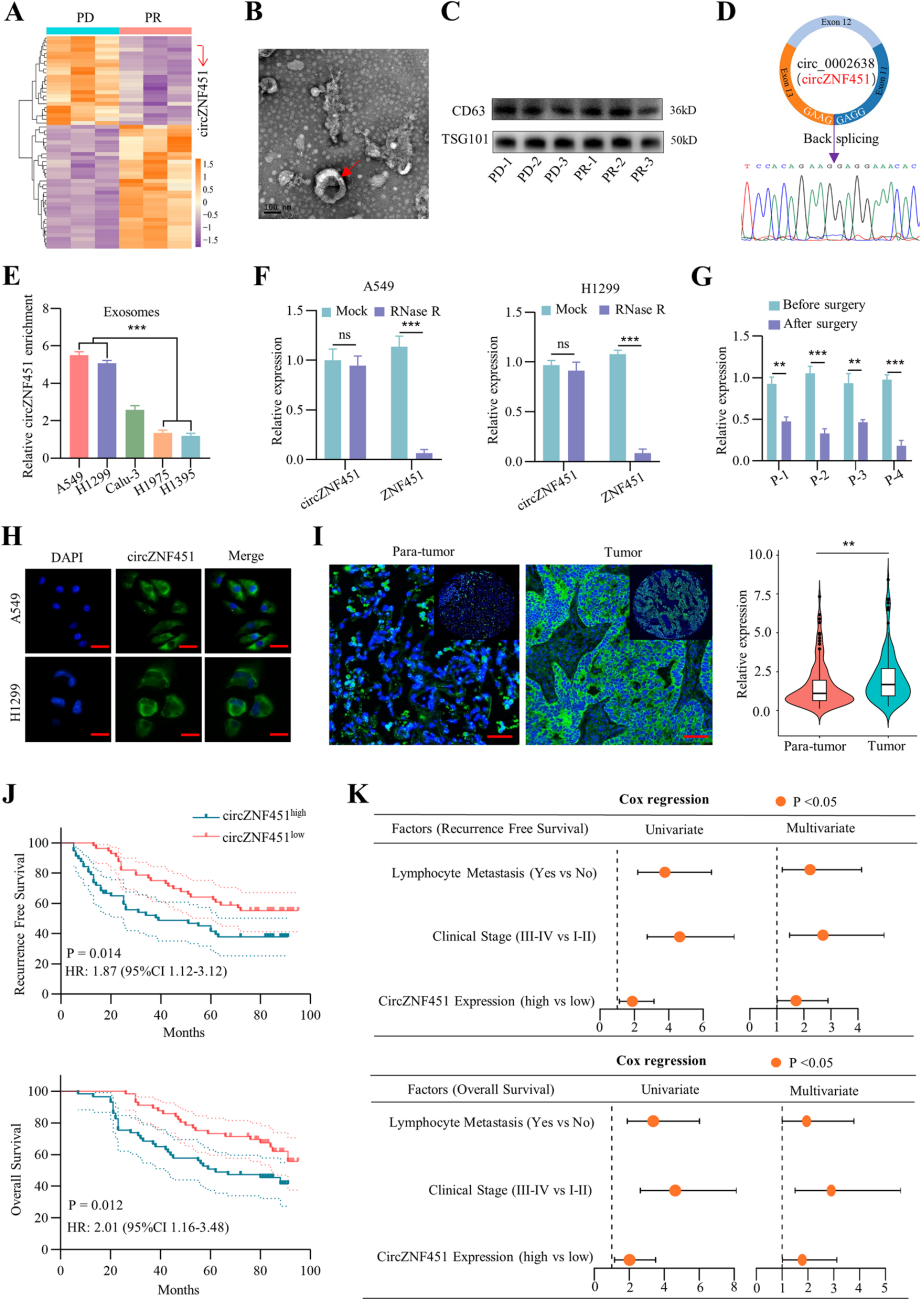
Clinical role of exosomal circZNF451 in immunotherapy and prognosis of LUAD patients. **A** Differential expression profiles of circRNAs (FC > 2 and *P* < 0.05) enriched in the peripheral blood of PR (*n* = 3) and PD (*n* = 3) patients receiving PD1 monotherapy (Opdivo) were measured by circRNA sequencing and are presented in a heat map. **B** Representative electron micrograph of exosomes detected in the sera of six LUAD patients receiving PD1 monotherapy; scale bar, 100 nm. **C** Exosome markers including TSG101 and CD63, in the exosomes of the six LUAD patients were detected by western blotting. **D** The back splicing site of circZNF451 (has-circ-0002638) was confirmed by Sanger sequencing. **E** Enrichment of circZNF451 in the exosomes of five LUAD cell lines (A549, H1299, Calu-3, H1975, and H1395) was measured by qRT-PCR. One-way ANOVA test was performed after adjusting for multiple comparisons. **F** After digestion with RNase R (10U) for 30 min, circZNF451 and ZNF451 stability in A549 and H1299 cells was measured by qRT-PCR. **G** Change in exosomal circZNF451 enrichment in four LUAD patients before and after surgery, as measured by qRT-PCR. **H** Localization of circZNF451 in A549 and H1299 cells as detected by FISH; scale bar, 50 μm. **I** Expression of circZNF451 was detected in a TMA containing tumor and paratumor tissues of 113 LUAD patients by FISH; scale bar, 100 μm. The paired Student’s *t* test was performed to analyze the difference. **J** Recurrence-free survival and overall survival of circZNF451^low^ and circZNF451^high^ groups in LUAD patients were analyzed by Kaplan–Meier and log-rank tests. **K** Factors that contribute to poor prognosis of the 113 LUAD patients according to the univariate and multivariate Cox regression analysis. All experiments with statistical analysis have been repeated for at least three times

To illustrate the potential role of exosomal circRNAs in immunotherapy resistance, the circRNA sequencing was performed to determine the level of circRNAs in peripheral blood from 6 LUAD patients treated with anti-PD1; of these, three patients were classified as progression disease (PD), and three patients were classified as partial response (PR) according to the RECIST1.1 evaluation (Supplementary Table 1) [14]. Fifty-five circRNAs exhibited differential abundance between PD and PR patients in the peripheral blood. Of these, 23 circRNAs were significantly upregulated in patients with PD, and 32 circRNAs were downregulated (FC_(PD/PR)_ > 2, *P* < 0.05). Figure 1A). Then, the exosomes in the sera of the six patients were extracted (Fig. 1B). The exosomal markers TSG101 and CD63 were verified by western blot (Fig. 1C) and the level of 4 most significantly changed circRNAs (FC > 4, *P* < 0.05) were detected via qRT-PCR. The results suggested that the level of has-circ-0002638 (circZNF451) was increased in the exosomes of PD patients compared to that in PR patients (Fig. S1A). Detection of circZNF451 in biopsied tumor tissues via qRT-PCR (*n* = 6) also showed that the expression of circZNF451 in exosomes and tumor tissues is highly consistent (Fig. S1B). Sanger sequencing identified the closed-loop structure of circZNF451 (Fig. 1D).

We further detected the expression of circZNF451 in LUAD cell lines and exosomes in the supernatants from corresponding cells via qRT-PCR. High level of circZNF451 was observed in A459 and H1299 cells, and low level was observed in H1395 and H1975 cells (Fig. 1E and S1C). Moreover, circZNF451 remained stable after incubation with RNase R (Fig. 1F). FISH staining of circZNF451 confirmed localization to the cytoplasm of different LUAD cell lines. We also separated the RNA from the nuclear and cytoplasm of H1395-circZNF451 and H1975-circZNF451 and found that the overexpression of circZNF451 was mainly detected in the cytoplasm (Fig. 1H and S1D). Importantly, we extracted the exosomes from the sera of 4 LUAD patients before and after surgery and identified the dramatically declined level of exosomal circZNF451 after the resection of malignant lesions (Fig. 1G). The situations of the four patients were presented in additional file 3.

We further examined the role of circZNF451 in the prognosis of LUAD patients. FISH staining of circZNF451 in a TMA containing 113 LUAD and peritumor tissues revealed increased circZNF451 levels in tumor tissues (Fig. 1I). Cox regression and Kaplan–Meier analyses established circZNF451 as an independent factor contributing to poor LUAD prognosis (Fig. 1J, K). Thus, our results suggested that the level of circZNF451 was elevated in the exosomes of advanced LUAD patients resistant to PD1 blockade and highlighted the potential influence of circZNF451 on the progression of LUAD.

### Exosomal circZNF451 reshapes the TME by inducing M2 polarization of macrophages and exhaustion of CD8^+^ T cells

**Fig. 2:**
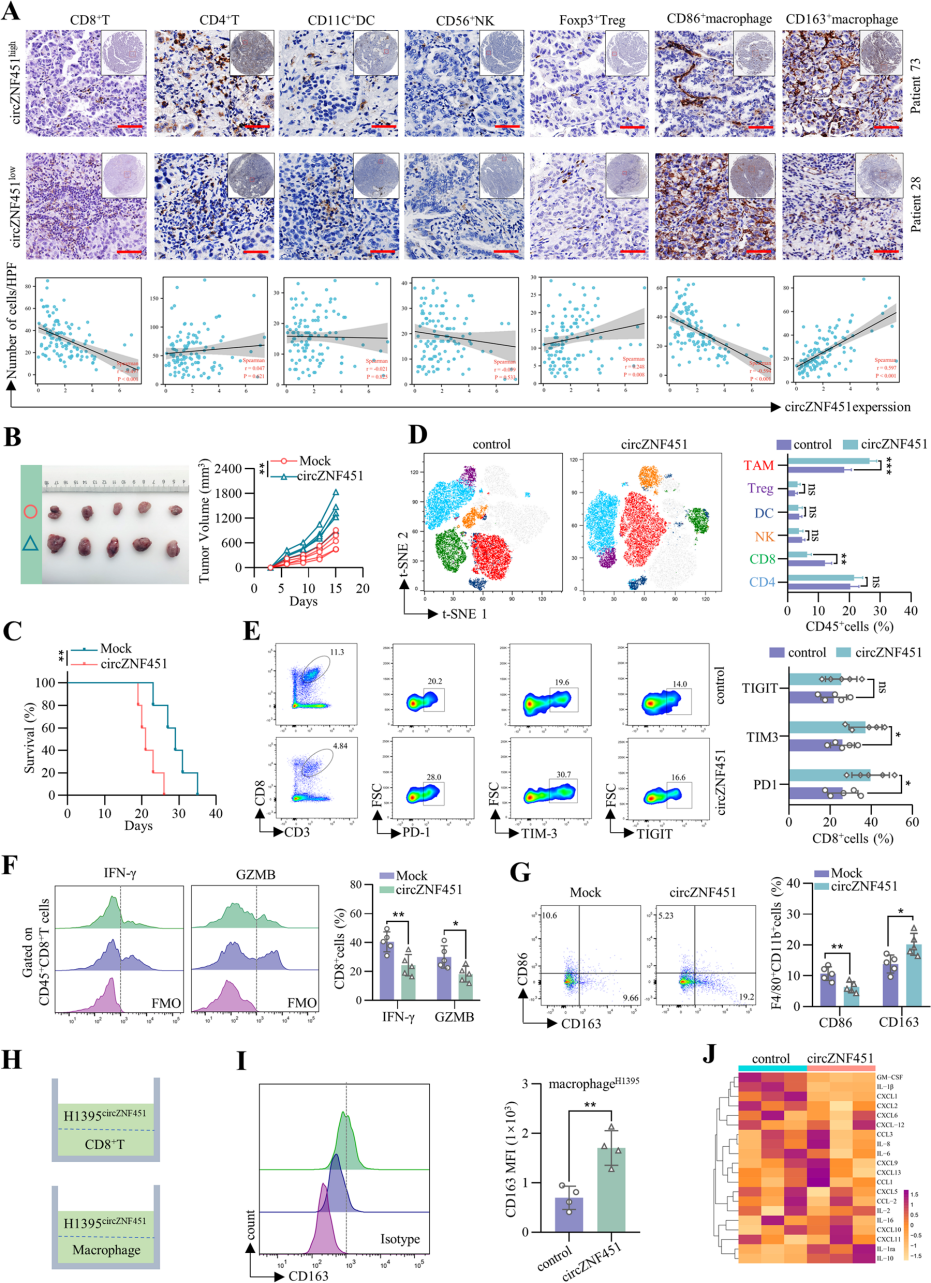
Exosomal circZNF451 reshapes the TME by inducing M2 polarization of macrophages and exhausting CD8^+^ T cells. **A** Infiltration of CD4^+^ T, CD8^+^ T, Foxp3^+^Treg, CD56^+^NK and CD11C^+^dendritic cells and CD86^+^ and CD163^+^ macrophages in the TMA containing the tumor tissues of 113 LUAD patients was analyzed by IHC; scale bar, 100 μm. A Spearman correlation analysis was applied. **B** LLC-circZNF451 and LLC-mock cells (5 × 10^6^) were subcutaneously implanted into C57BL/6 J mice (*n* = 5 per group). Tumor volumes were measured every 3 days for 2 weeks. Tumor volume equals 1/2 length × width^2^. **C** LLC-circZNF451 and LLC-mock cells were subcutaneously implanted into C57BL/6 J mice (*n* = 5 per group), and survival was recorded. The Kaplan–Meier and log-rank tests were used for comparison. **D** After subcutaneous implantation of LLC-circZNF451 and LLC-mock cells, immune infiltration profiles, including CD8^+^ T cells, CD4^+^ T cells, Treg cells, NK cells, DCs, and TAMs, in the tumor tissue were detected by flow cytometry. **E** Immune checkpoints, including PD1, TIM3, and TIGIT, in CD8^+^ T cells were detected by flow cytometry in the circZNF451 and WT groups. **F** Cytotoxic activities of CD8^+^ T cells, including IFN-γ and GZMB, were detected by flow cytometry in the circZNF451 and WT groups. **G** Infiltration of M1 (F4/80^+^CD11b^+^CD86^+^) and M2 (F4/80^+^CD11b^+^CD163^+^) polarized macrophages in tumor tissues of circZNF451 and WT groups was detected by flow cytometry. **H** Diagram of the coculture model. H1395-circZNF451/control cells (5 × 10^5^) were cocultured with macrophages (1 × 10^6^; PMA stimulated THP-1 cells for 24 h), or CD8^+^ T cells (1 × 10^5^) from healthy donors for 48 h. **I** The M2 phenotype of macrophages (CD163^+^) after coculture with H1395-circZNF451 cells for 48 h was detected by flow cytometry. **J** Cytokine and chemokine profiles in the supernatant of LPS-stimulated macrophages were measured by the human cytokine/chemokine microarray after coculturing with H1395-control and H1395-circZNF451 cells for 48 h. **B**, **D**, **E**–**G** and **I** were analyzed using a two-tailed, Student’s *t* test. All experiments with statistical analysis have been repeated for at least three times

To further explore the potential impacts of circZNF451 on the TME, the IHC staining of CD4^+^T, CD8^+^T, CD56^+^NK, Foxp3^+^Treg, CD11C^+^dendritic cell (DC), CD163^+^macrophage and CD86^+^macrophage was performed in the 113 LUAD tissues. We found that the infiltration of CD8^+^T and CD86^+^macrophage was exhausted while the infiltration of Foxp3^+^Treg and CD163^+^macrophage was increased in circZNF451^high^ group (Fig. S2A). The expression of circZNF451 was positively correlated with the infiltration of CD163^+^ macrophage but negatively correlated with that of CD8^+^ T and CD86^+^ macrophage (Fig. 2A). To further verify these results, we constructed the LLC-circZNF451 cell lines (Fig. S2B) and an in vivo model in immunocompetent mice (C57BL/6 J) was developed with subcutaneous implantation of LLC-circZNF451 cells. CircZNF451 accelerated growth of subcutaneous tumors and lead to shorter survival in mice (Fig. 2B, C). Flow cytometry analysis confirmed the exhaustion of CD8^+^ T cells and increased infiltration of TAMs in tumors with circZNF451 overexpression (Fig. 2D). CircZNF451 also induced conversion of cytotoxic lymphocytes to exhausted CD8^+^ T cells, with evidence of increased expression of inhibitory immune checkpoint PD1 and TIM3 and decreased secretion of cytotoxic cytokines interferon-γ (IFN-γ) and granzyme B (GZMB; Fig. 2E, F). Additionally, we discovered increased infiltration of CD163^+^ macrophages and exhaustion of CD86^+^ macrophages after circZNF451 overexpression (Fig. 2G), suggesting that exosomal circZNF451 might elicit M2 polarization of macrophages.

Furthermore, we investigated the impacts of exosomal circZNF451 enrichment on macrophages and CD8^+^ T cells and constructed a coculture model by culturing LUAD cell lines with CD8^+^ T cells or macrophages (PMA-stimulated THP-1 cells; Fig. 2H). The circZNF451 was silenced in A549 and H1299 (high level of endogenous circZNF451) and overexpressed in H1395 and H1975 (low level of endogenous circZNF451, Fig. S2C). Coculture of H1395-circZNF451 cells and CD8^+^ T cells from healthy donors did not impair the secretion of IFN-γ and GZMB in CD8^+^ T cells (Fig. S2D), but coculture of H1395-circZNF451 cells and LPS-stimulated macrophages induced the enhanced expression of M2 polarization markers CD163, Arg1 and IL-10 (Fig. 2I and S2E). The qRT-PCR showed enhanced level of circZNF451 in macrophages after co-culturing with H1395-circZNF451 cells while the administration of exosome inhibitor GW4869 (20 μM) abolished the enrichment (Fig. S2F). The administration of the GW4869 in the coculture model also abolished M2 polarization of macrophages (Fig. S2G, H). Moreover, the supernatant from the macrophage/H1395-circZNF451 cells induced the dysfunction of cytotoxic CD8^+^T cells (Fig. S2I). The CFSE assay also confirmed the impaired proliferation of CD8^+^ T cells cultured by the supernatant from the macrophage/H1395-circZNF451 cells (Fig. S2J). We further identified the above results by coculturing macrophages with A549. Similarly, the knockdown of circZNF451 in A549 diminished the M2 polarization of macrophages, which also rescued the dysfunction and proliferation of CD8^+^ T cells (Fig. S2K-N). In addition, cytokines and chemokines in the supernatant of the coculture model were measured with a Luminex human cytokine/chemokine microarray, and upregulation of anti-inflammatory cytokines, including IL-10 and IL-1Ra, and downregulation of proinflammatory cytokines, including IL-1β, GM-CSF (CSF2), and CXCL1, were identified after coculturing with H1395-circZNF451 cells (Fig. 2J). These results demonstrate that circZNF451 can induce M2 polarization of macrophages, which further elicits the dysfunction of CD8^+^ T cells and resets an immune-suppressed TME.

### Exosomal circZNF451 targets macrophages to interact with and enhance FXR1 ubiquitination

**Fig. 3:**
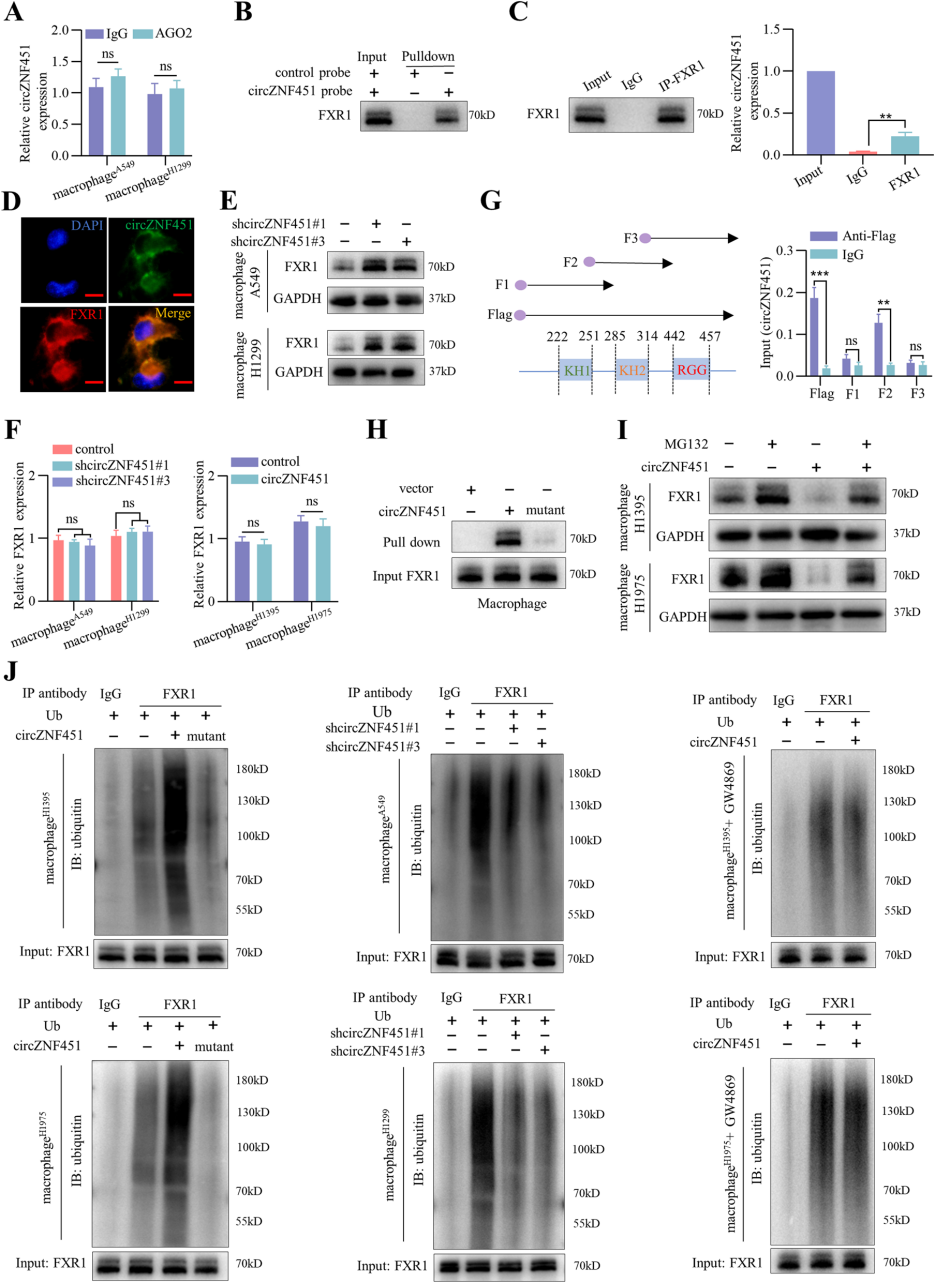
Exosomal circZNF451 interacts with and enhances FXR1 ubiquitination in macrophages. **A** After coculturing with H1299 and A549 cells (5 × 10^5^) for 48 h, the RIP assay was performed in macrophages (1 × 10^6^; PMA stimulated THP-1 cells for 24 h) with IgG and AGO2 antibody. **B** After coculturing with A549 for 48 h, RNA pulldown assay was performed with the biotinylated circZNF451 probe to detect the interaction with FXR1 in macrophage. **C** After coculturing with A549 for 48 h, RIP assay was performed in macrophage to confirm the interaction between circZNF451 and FXR1 with the FXR1 antibody. **D** Colocalization of circZNF451 and FXR1 in macrophage was detected via FISH; scale bar, 50 μm. **E** After coculturing with A549/H1299-shcircZNF451 cells, FXR1 expression in macrophages (1 × 10^6^) was detected via western blotting. **F** After coculturing with A549/H1299-shcircZNF451 and H1395/H1975-circZNF451 cells (5 × 10^5^), FXR1 expression in macrophages (1 × 10^6^) was detected via qRT-PCR. **G** Vectors encoding three fragments of FXR1 containing the three RNA-binding domains (KH1, KH2, and RGG box) with circZNF451 were transfected into 293 T cells, and RIP was performed with IgG and Flag antibody to detect circZNF451 enrichment. **H** Interaction between FXR1 and circZNF451 in macrophage was verified by RNA pulldown with biotinylated circZNF451 WT and mutant probes. **I** After administration of MG132 (20 μM) for 48 h, FXR1 expression in macrophages cocultured with H1395/H1975-circZNF451 was detected by western blotting. **J** IP was performed to measure FXR1 ubiquitination after coculturing macrophages with LUAD cell lines with silenced (A549 and H1299) or overexpressed (H1395 and H1975) circZNF451 and GW4869 administration (20 μM) for 48 h. **A**, **C** and **G** used the two-tailed, unpaired Student’s *t* test. **F** was analyzed by one-way ANOVA test after adjusting for multiple comparisons. All experiments with statistical analysis have been repeated for at least three times

CircRNAs can act as microRNA (miRNA) sponges. However, a RIP assay showed that circZNF451 could not be enriched by AGO2, which suggests that circZNF451 may not function as a miRNA sponge (Fig. 3A). Thus, we further tested whether circZNF451 function by interacting with proteins. An RNA pulldown assay was performed with a biotinylated circZNF451 probe in macrophages, and the silver staining showed a specific band at around 70 kDa (Fig. S3A). Mass spectrometry analysis of the band showed that the top three enriched proteins were HNRNPM, FXR1, and EIF3L, and AGO2 was not included (Additional File 1). The mass spectrometry data was further overlapped with the predicted candidates in circInteractome and the most detected RNA binding motifs in catRAPID database, which strongly suggested FXR1 as the downstream of circZNF451 (Fig. S3B). RNA pulldown and RIP assays further confirmed the specific interaction between circZNF451 and FXR1 (Fig. 3B, C). After coculturing with A549 cells for 48 h, RNA FISH and immunofluorescence staining of circZNF451 and FXR1 confirmed colocalization in the cytoplasm of macrophages (Fig. 3D). Furthermore, coculture with A549-shcircZNF451 and H1299-shcircZNF451 cells enhanced FXR1 protein levels in macrophages (PMA-stimulated THP-1 cells), while coculture with H1395-circZNF451 and H1975-circZNF451 cells showed the opposite trend, which was reversed after GW4869 administration (20 μM; Fig. 3E and S3C). Nonetheless, FXR1 mRNA expression did not change (Fig. 3F), suggesting that circZNF451 regulates expression of FXR1 protein.

FXR1 contains two KH domain (KH1 and KH2) and one RGG box, which are responsible for the interaction with RNAs [15]. To identify the exact RNA-binding domain for circZNF451, we constructed three Flag-tagged vectors encoding the fragments of FXR1. After co-transfection with circZNF451 into HEK293T cells, RIP assay showed that KH2 was mainly responsible for binding to circZNF451 (Fig. 3G). Here, we also explored the binding motif of FXR1 in the RBPsuite database and identified one potential binding region for FXR1 in the circZNF451 sequence (Fig.... S3D). We therefore mutated the FXR1-binding site in circZNF451 (Fig. S3E), which dramatically impaired the interaction between circZNF451 and FXR1 in macrophage (Fig. 3H).

Regulation of FXR1 protein rather than mRNA expression by circZNF451 indicates circZNF451 may destabilize FXR1 protein in macrophages. Indeed, circZNF451 overexpression downregulated FXR1 protein levels, administration of the protease inhibitor MG132 partially restored it (Fig. 3I). Overexpression of circZNF451 significantly enhanced FXR1 ubiquitination, which was reversed by circZNF451 mutation or knockdown. Administration of GW4869 (20 μM) also abolished the enhanced ubiquitination of FXR1 induced by circZNF451 (Fig. 3J). Together, these results suggest that LUAD-derived exosomal circZNF451 may drive FXR1 ubiquitination in macrophages.

### circZNF451 enhances TRIM56 mediating FXR1 ubiquitination

**Fig. 4:**
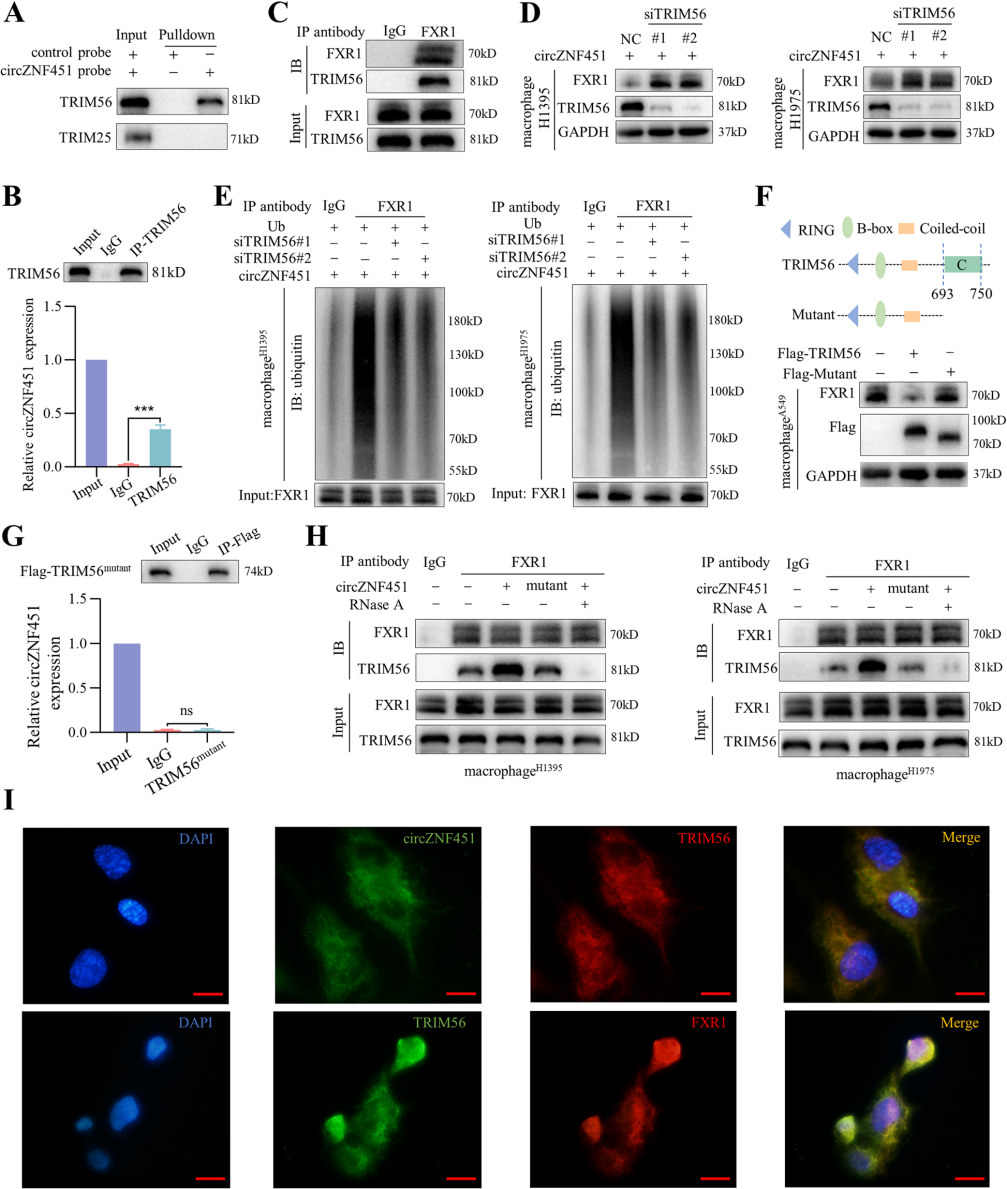
circZNF451 enhances the E3 ligase TRIM56 mediating FXR1 ubiquitination. **A** After coculturing with A549 for 48 h, RNA pulldown was performed in macrophages (PMA stimulated THP-1 cells for 24 h) to verify the interaction between circZNF451 and TRIM56 and TRIM25. **B** After coculturing with A549 for 48 h, RIP was performed in macrophages to confirm the interaction between circZNF451 and TRIM56. **C** After coculturing with A549 for 48 h, Co-IP was performed in macrophages to verify the interaction between FXR1 and TRIM56. **D** After coculturing with H1395/H1975-circZNF451, FXR1 expression in macrophage-siTRIM56 was measured by western blotting. **E** IP was performed to detect FXR1 ubiquitination in macrophage-siTRIM56 after coculturing with H1395/H1975-circZNF451 for 48 h. **F** After mutation of TRIM56 (deletion in the C terminus), FXR1 expression in macrophages (transfected with Flag-TRIM56 or Flag-TRIM56^mutant^) was detected via western blotting after coculturing with A549 for 48 h. **G** After coculturing with A549 for 48 h, RIP was performed in macrophages transfected with TRIM56^mutant^ to confirm the interaction between circZNF451 and TRIM56^mutant^. **H** After digestion by RNase A (10U) for 30 min, co-IP was used to verify the interaction between FXR1 and TRIM56 in macrophage after coculturing with H1395/H1975-circZNF451 cells for 48 h. **I** Localization of circZNF451, FXR1, and TRIM56 in macrophage was detected by FISH and immunofluorescence; scale bar, 50 μm. **B** and **G** used the two-tailed, unpaired Student’s *t* test. All experiments with statistical analysis have been repeated for at least three times

To identify the ubiquitinating enzyme for FXR1, we reanalyzed the mass spectrometry data and identified two E3 ligases, TRIM56 and TRIM25. RNA pulldown and RIP confirmed that circZNF451 could specifically interacted with TRIM56 (Fig. 4A, B). We further performed a Co-IP assay in macrophages (PMA-stimulated THP-1 cells) and found that FXR1 could bind TRIM56 after coculturing with A549 for 48 h (Fig. 4C). Knockdown of TRIM56 in macrophages abolished the reduction of FXR1 mediated by H1395/H1975-circZNF451(Fig. 4D). Consistently, ubiquitination of FXR1 was weakened following TRIM56 knockdown (Fig. 4E).

The C terminus (amino acids 693–750) of TRIM56 is a region critical for RNA binding [16]. Thus, we constructed a Flag-tagged mutant TRIM56 with the deletion of C-terminal and transfected it into macrophages. After coculture with A549 cells, Western blotting showed that the transfection of flag-TRIM56 enhanced the downregulation of FXR1 while the mutation of TRIM56 rescued it (Fig. 4F). RNA pulldown and RIP assays showed that mutation of TRIM56 abolished the interaction between circZNF451 and TRIM56 in macrophage (Fig. 4G and S4A). We also observed that mutation in circZNF451 and digestion with RNase A impeded the interaction between TRIM56 and FXR1 in macrophages, which were consistent with the results confirmed by the knockdown of circZNF451 in A549 and H1299 (Fig. 4H and S4B). RNA FISH and immunofluorescence showed that circZNF451, FXR1, and TRIM56 were mainly presented in the cytoplasm of macrophages (Fig. 4I). These data suggest that circZNF451 functions as a scaffold to enhance the interaction between TRIM56 and FXR1 proteins in macrophages.

### The circZNF451 regulates the phenotype of macrophages via complex with TRIM56 –FXR1

**Fig. 5:**
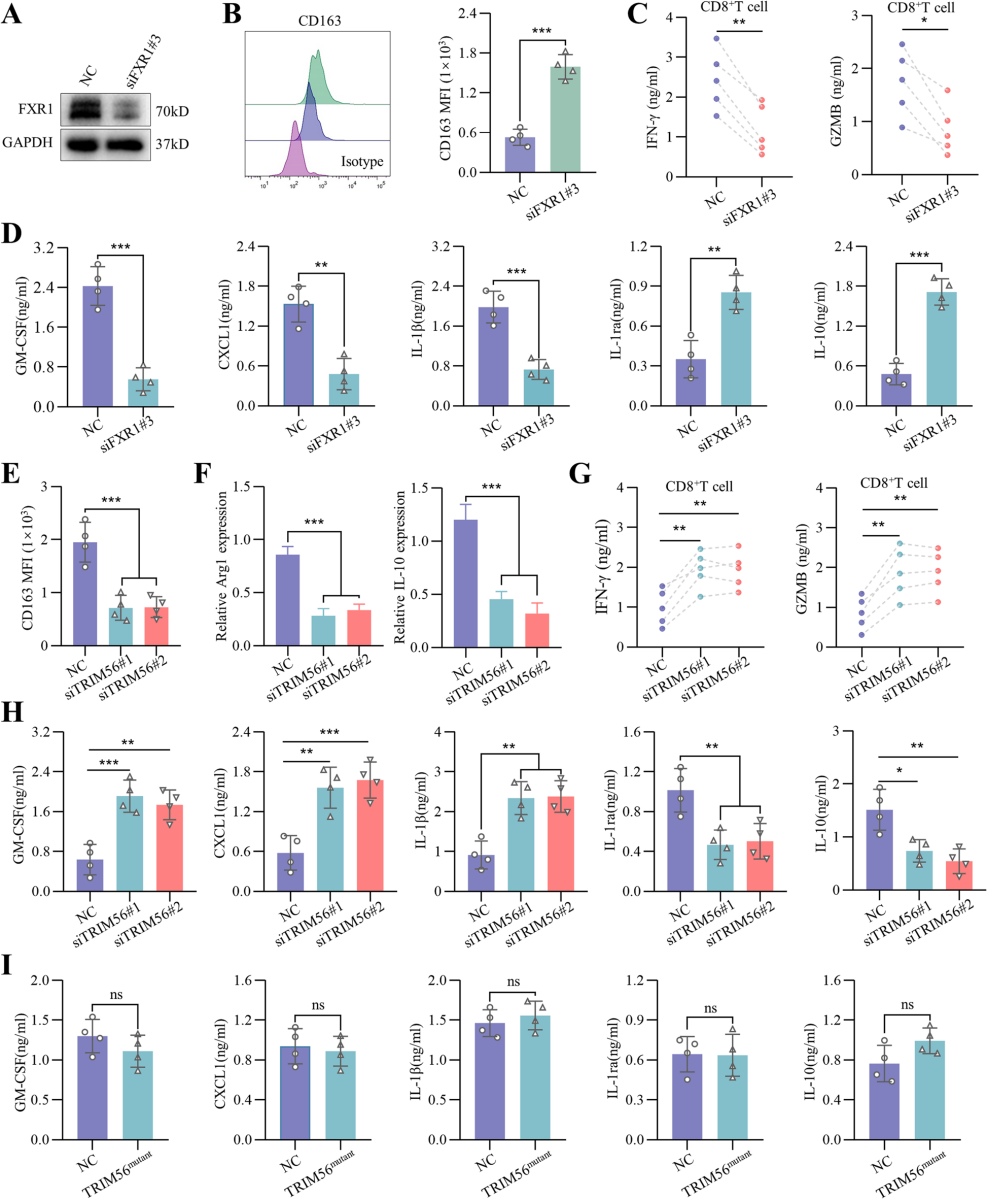
The circZNF451–TRIM56–FXR1 complex regulates the immune phenotype of macrophages. **A** Silencing of FXR1 in macrophages (PMA stimulated THP-1 cells for 24 h) with the siRNA was verified by western blotting. **B** The impact of FXR1 knockdown on the M2 phenotype of LPS-stimulated macrophages was detected by flow cytometry. **C** Concentrations of IFN-γ and GZMB in the supernatant of CD8^+^ T cells cocultured with the supernatant from macrophage-siFXR1 for 48 h were detected by ELISA. **D** After LPS stimulation, concentrations of GM-CSF, CXCL1, IL-1β, IL-1Ra, and IL-10 in the supernatant of macrophage-siFXR1 were measured by ELISA. **E** The impact of TRIM56 knockdown on the M2 phenotype of LPS-stimulated macrophages after coculturing with H1395-circZNF451 for 48 h was analyzed by flow cytometry. **F** The M2 phenotype markers Arg1 and IL-10 were measured by qRT-PCR after silencing TRIM56 in LPS-stimulated macrophages cocultured with H1395-circZNF451 for 48 h. **G** Concentrations of IFN-γ and GZMB in the supernatant of CD8^+^ T cells cocultured with the supernatant from the macrophage-siTRIM56/H1395-circZNF451 for 48 h were detected by ELISA. **H** Concentrations of GM-CSF, CXCL1, IL-1β, IL-1Ra, and IL-10 in the supernatants of H1395-circZNF451/macrophage-siTRIM56 stimulated by LPS were measured by ELISA. **I** After LPS stimulation, concentrations of GM-CSF, CXCL1, IL-1β, IL-1Ra, and IL-10 in the supernatants of macrophage-TRIM56^mutant^/A549 cells were measured by ELISA. **B**-**D** and **I** used the two-tailed, Student’s *t* test. **E**–**H** were analyzed by one-way ANOVA test after adjusting for multiple comparisons. All experiments with statistical analysis have been repeated for at least three times

Interactions between circZNF451, FXR1, and TRIM56 indicate the impacts of the FXR1**–**circZNF451**–**TRIM56 complex on the macrophage immune phenotype. We first knocked down FXR1 in macrophage (PMA-stimulated THP-1 cells) with short interfering RNA (Fig. 5A). After LPS stimulation, flow cytometry analysis showed that reduction of FXR1 leads to M2 polarization of macrophages (Fig. 5B and S4C). Supernatant from macrophage-siFXR1 cells further induced cytotoxic dysfunction of CD8^+^ T cells, with decreased secretion of IFN-γ and GZMB (Fig. 5C). We also detected cytokines in the supernatant via ELISA and observed a significant upregulation of anti-inflammatory cytokines (e.g., IL-1Ra and IL-10) and a reduction in pro-inflammatory cytokines (e.g., GM-CSF, CXCL1, and IL-1β) after silencing FXR1 in macrophages (Fig. 5D).

By contrast, silencing TRIM56 in macrophage inhibited M2 polarization after coculturing with H1395-circZNF451 cells (Fig. 5E, F), and supernatant from the coculture system rescued the cytotoxic function of CD8^+^ T cells inhibited by circZNF451 overexpression (Fig. 5G). Additionally, GM-CSF, CXCL1, and IL-1β levels were elevated in the supernatant, while IL-1Ra and IL-10 levels were dramatically reduced after silencing TRIM56 in macrophages (Fig. 5H). However, overexpression of mutant TRIM56 had no impact on M2 polarization and production of pro- or anti-inflammatory cytokines in macrophages after coculture with A549 cells (Fig. 5I and S4D, E).

### Degradation of FXR1 activates the ELF4–IRF4 pathway in macrophages

**Fig. 6:**
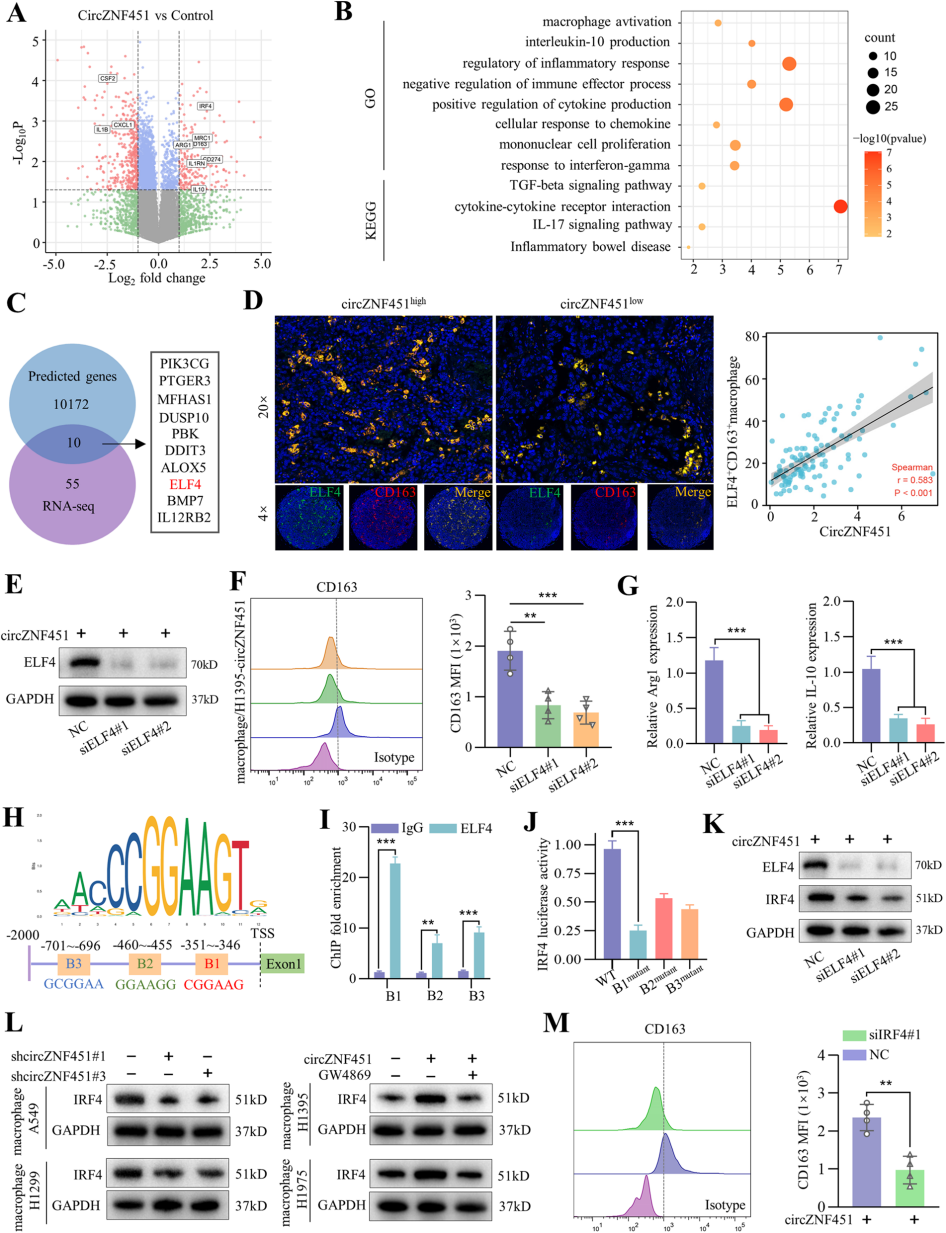
Degradation of FXR1 activates the ELF4–IRF4 pathway in macrophages. **A** Volcano map of mRNA expression in macrophages (PMA stimulated THP-1 cells for 24 h) cultured with H1395-circZNF451 and control cells. **B** GO and KEGG analysis of the differently expressed candidates (FC > 2, *P* < 0.05) that may be regulated by circZNF451 in macrophages. **C** Overlap of immune-related candidates in the differently expressed genes (*n* = 65) identified by RNA-seq and the predicted candidates for FXR1 in ENCORI (*n* = 10,182). **D** Dual immunofluorescence staining of ELF4 and CD163 in the TMA containing the tumor tissues of 113 LUAD patients and the correlation of ELF4^+^CD163^+^ macrophages with circZNF451 expression was explored by Spearman correlation analysis; scale bar, 100 μm. **E** Silencing of ELF4 in macrophages was verified by western blotting. **F** After coculturing with H1395-circZNF451 for 48 h, the influence of ELF4 silencing in LPS-stimulated macrophages on the M2 phenotype was confirmed by flow cytometry. **G** After coculturing with H1395-circZNF451 for 48 h, the M2 phenotype markers Arg1 and IL-10 were measured by qRT-PCR after ELF4 knockdown in macrophages stimulated by LPS. **H** The binding motif of ELF4 and three putative binding sites in the promoter region of IRF4. **I** A ChIP assay was performed in macrophage to verify the binding region of ELF4 in IRF4. **J** A luciferase assay was performed to confirm the binding of ELF4 in the three putative regions of IRF4. **K** IRF4 expression was detected in LPS-stimulated macrophage-siELF4 cells via western blotting after coculturing with H1395-circZNF451 cells for 48 h. **L** IRF4 expression in LPS-stimulated macrophages was measured by western blotting after coculturing with LUAD cell lines with silencing (A549 and H1299) or overexpression (H1395 and H1975) of circZNF451 and administration of GW4869 (20 μM) for 48 h. **M** The M2 phenotype of LPS-stimulated macrophage-siIRF4 cells was detected by flow cytometry after coculturing with H1395-circZNF451 cells for 48 h. **F**, **G** and **J** were analyzed by one-way ANOVA test after adjusting for multiple comparisons. **I** and **M** used the two-tailed, Student’s *t* test. All experiments with statistical analysis have been repeated for at least three times

The RBP FXR1 regulates the stability of downstream mRNAs [17, 18]. Thus, RNA sequencing was performed to detect the profiles of mRNAs in macrophages (PMA-stimulated THP-1 cells) after coculture with H1395-circZNF451 and H1395-control cells. We found that the macrophages co-cultured with H1395-circZNF451 and stimulated by LPS upregulated ARG1, MRC1 (CD206), CD163, CD274 (PD-L1), IL-Ra (IL-1RN) and IL-10, while the expression of GM-CSF (CSF2), CXCL1, and IL-β was reduced (Fig. 6A). KEGG and GO analysis revealed a significant change in immune-related responses (Fig. 6B). Further analysis revealed that 65 immune-related genes among the identified differently expressed mRNAs overlapped with the predicted candidates of FXR1 in the ENCORI database, and 10 potential candidates were identified to be the downstream of FXR1 in macrophages. Because ELF4 was demonstrated the most significant shift in expression among the 10 candidates, and previous study identified a critical role for ELF4 in regulating inflammatory responses [19], we speculated that FXR1 degradation might enhance the anti-inflammatory phenotype of macrophages via ELF4 (Fig. 6C, Additional File 2). Previous studies reported the binding of 3’UTR was important for FXR1 to promote the degradation of the target genes. Thus, we constructed the vectors containing the 3’UTR of ELF4 and transferred it into the macrophages. The dual-luciferase reporter assay identified that the relative luciferase activity was obviously weakened while the co-transfection with the siFXR1#3 partially restored it, which confirmed that FXR1 could bind the 3’UTR of ELF4 to regulate its stability (Figure S4F). Interestingly, we found that both the overexpression of circZNF451 in LUAD and silencing of FXR1 in macrophages could contribute to enhanced expression of ELF4 (Fig. S5A, B). Also, immunofluorescence staining of ELF4 and CD163 in the TMA suggested a positive correlation between the expression of circZNF451 and infiltration of ELF4^+^CD163^+^ macrophages (Fig. 6D).

Here, we further identify the impact of ELF4 on M2 polarization of macrophages. By interfering the ELF4 in macrophages, we found that the reduction of ELF4 dramatically abrogated M2 polarization endowed by H1395- circZNF451 (Fig. 6E–G). Silencing of ELF4 also reduced the production of IL-1Ra and IL-10, but increased the secretion of GM-CSF, CXCL1, and IL-1β in the macrophage supernatant (Fig. S5C).

Analysis of ChIP-seq data from a published study [20] revealed that recruitment of ELF4 led to a significant induction of interferon regulatory factors (IRFs), including IRF4, which was significantly increased in our RNA-seq data (Fig. 6A). Previous studies had identified IRF4 as an important mediator of M2 polarization of macrophages [21], suggesting that ELF4 could elicit M2 polarization of macrophages via activation of IRF4. We identified the ELF4 binding motif in JASPAR and found three putative binding sites, B1 (− 351 to − 346), B2 (− 460 to − 455), and B3 (− 701 to − 696), with the core sequence of GGAA within a 2,000-bp distance from the IRF4 transcription start site (Fig. 6H). The ChIP assay showed that B1 had the highest binding affinity with ELF4 (Fig. 6I). We next constructed vectors of the promoter containing the mutation of the three binding sites (GGAA to AAAA), and luciferase activities in HEK293T cells were most significantly inhibited in the vectors containing the mutant sequence in B1 (Fig. 6J). Knockdown of ELF4 also decreased IRF4 expression in macrophages at both the mRNA and protein levels after coculture with H1395-circZNF451 cells (Fig. 6K and S5D). Moreover, knockdown of circZNF451 in LUAD cell lines decreased IRF4 expression in macrophage, while overexpression of circZNF451 showed the opposite trend, which was reversed by administration of GW4869 (Fig. 6L and S5E). Silencing of IRF4 also inhibited M2 polarization in macrophages cocultured with H1395-circZNF451 (Fig. 6M). Consistently, macrophage cytokine levels followed similar patterns as the knockdown of ELF4 (Fig. S5F). Collectively, these results suggest that degradation of FXR1 by circZNF451 activates the ELF4–IRF4 pathway in macrophages.

### Conditional knockout of ELF4 in macrophages enhances immunotherapy efficacy in a xenograft model with overexpressed circZNF451

**Fig. 7:**
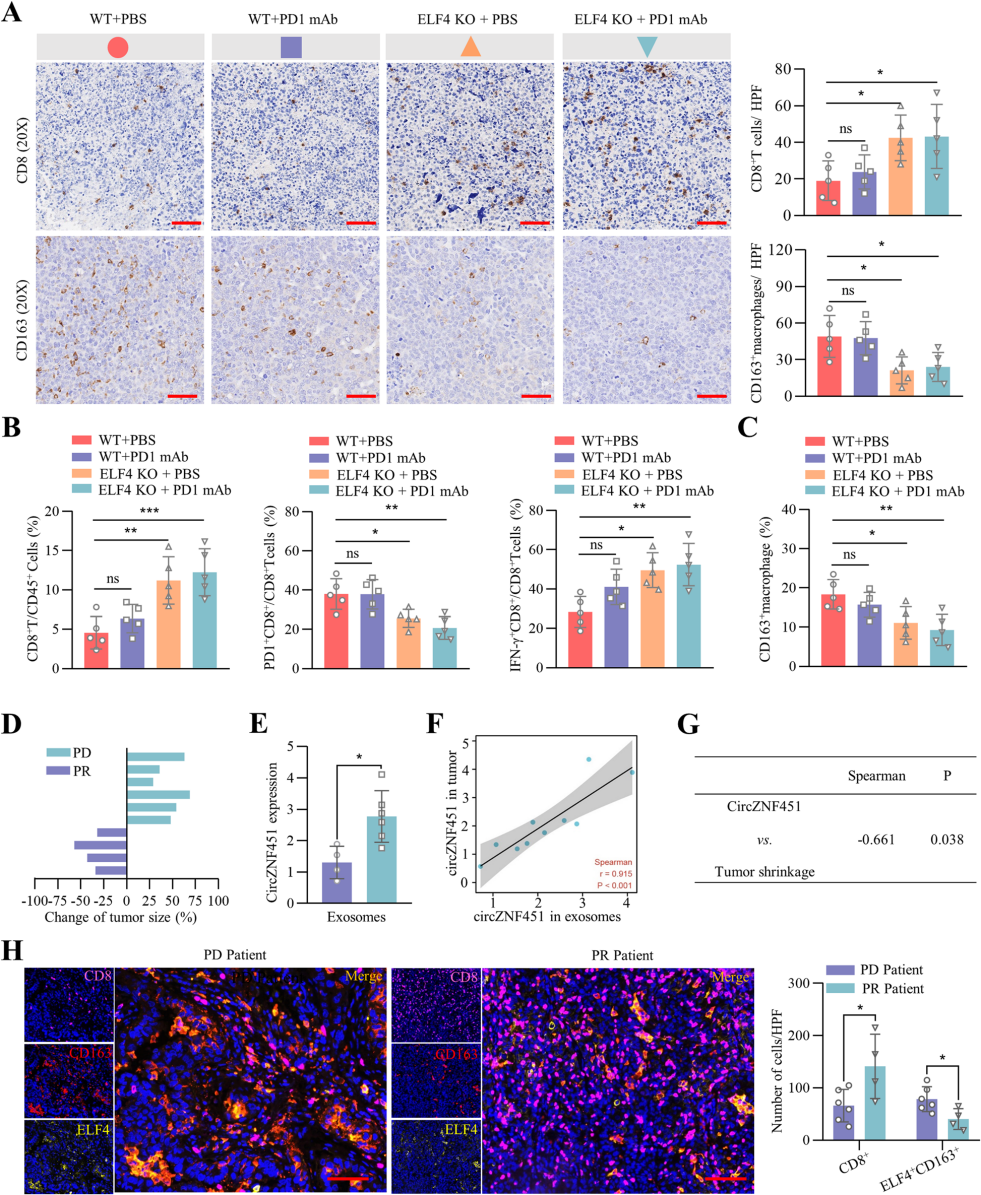
Conditional knockout of ELF4 in macrophages enhances immunotherapy efficacy in a xenograft model with overexpressed circZNF451. **A** Infiltration of CD8^+^ T cells and CD163^+^ macrophages in the tumor tissues of the four groups (WT; WT + anti-PD1; ELF4 KO and ELF4 KO + PD1) was verified by IHC (n = 5 for each group). **B** Infiltration of CD8^+^ T cells in the TME; the percentage of PD1^+^/IFN-γ^+^CD8^+^ T cells of the four groups were detected by flow cytometry. **C** Infiltration of CD163^+^ macrophages in the TME of the four groups was analyzed by flow cytometry. **D** Change in tumor size in the ten LUAD patients (six PD and four PR) receiving PD1 blockade monotherapy. **E** Enrichment of circZNF451 in the exosomes in four PR and six PD patients receiving PD1 blockade monotherapy was verified by qRT-PCR. **F** Correlation between the enrichment of circZNF451 in exosomes and the expression of circZNF451 in tumor tissues of the 10 LUAD patients receiving PD1 blockade monotherapy. A Spearman correlation analysis was applied. **G** Correlation between the enrichment of circZNF451 in exosomes and tumor shrinkage was analyzed by Spearman correlation analysis. **H** Infiltration of CD8^+^ T cells and ELF4^+^CD163^+^ macrophages in the tumor tissues of 10 patients receiving PD1 blockade monotherapy; scale bar, scale bar, 100 μm. **A**-**C** were analyzed by one-way ANOVA test after adjusting for multiple comparisons. **H** used the two-tailed, unpaired Student’s *t* test. All experiments with statistical analysis have been repeated for at least three times

Considering the potential regulation of ELF4 on the phenotype of macrophages, we speculated that ELF4 knockout might rescue the immune-suppressed TME induced by exosomal circZNF451 and enhance sensitivity to PD1 blockade. Thus, we constructed a mouse model with conditional knockout of ELF4 in macrophages via C57BL/6 J-Lyz2^CreERT2^ and C57BL/6 J-ELF4^em1(flox)Smoc^ mice, which were subcutaneously implanted with LLC-circZNF451 cells. PD1 blockade was administered 7 days later at an interval of 3 days. Knockout of ELF4 in macrophages dramatically enhanced the antitumor effect of PD1 blockade and inhibited tumor growth (Fig. S6A). The IHC staining showed that PD1 blockade enhanced CD8^+^ T cell infiltration and led to the exhaustion of CD163^+^ macrophages in ELF4-knockout mice with overexpression of circZNF451 (Fig. 7A). Consistently, the flow cytometry analysis also confirmed the similar results and identified the recovery of cytotoxic CD8^+^ T cell after the administration of PD1 blockade in ELF4-knockout mice with overexpression of circZNF451 (Fig. 7B, C and S6B).

To further verify the impacts of exosomal circZNF451 on the TME and immunotherapy, we selected ten LUAD patients receiving PD1 blockade (Opdivo) treatment, in whom six were evaluated as PD and four were evaluated as PR (Fig. 7D and Supplementary Table 6). Sera and biopsied tumor tissues of the ten patients were collected for analysis. Enrichment of circZNF451 in exosomes was significantly higher in PD patients and was consistent with the expression of circZNF451 in paired tumor tissue (Fig. 7E, F), and enrichment of circZNF451 in exosomes was negatively associated with tumor shrinkage (Fig. 7G). The mIF staining of CD8^+^ T cells and ELF4^+^CD163^+^ macrophages showed high infiltration of ELF4^+^CD163^+^ macrophages and low infiltration of CD8^+^ T cells in the tumor tissues of PD patients (Fig. 7H).

**Fig. 8:**
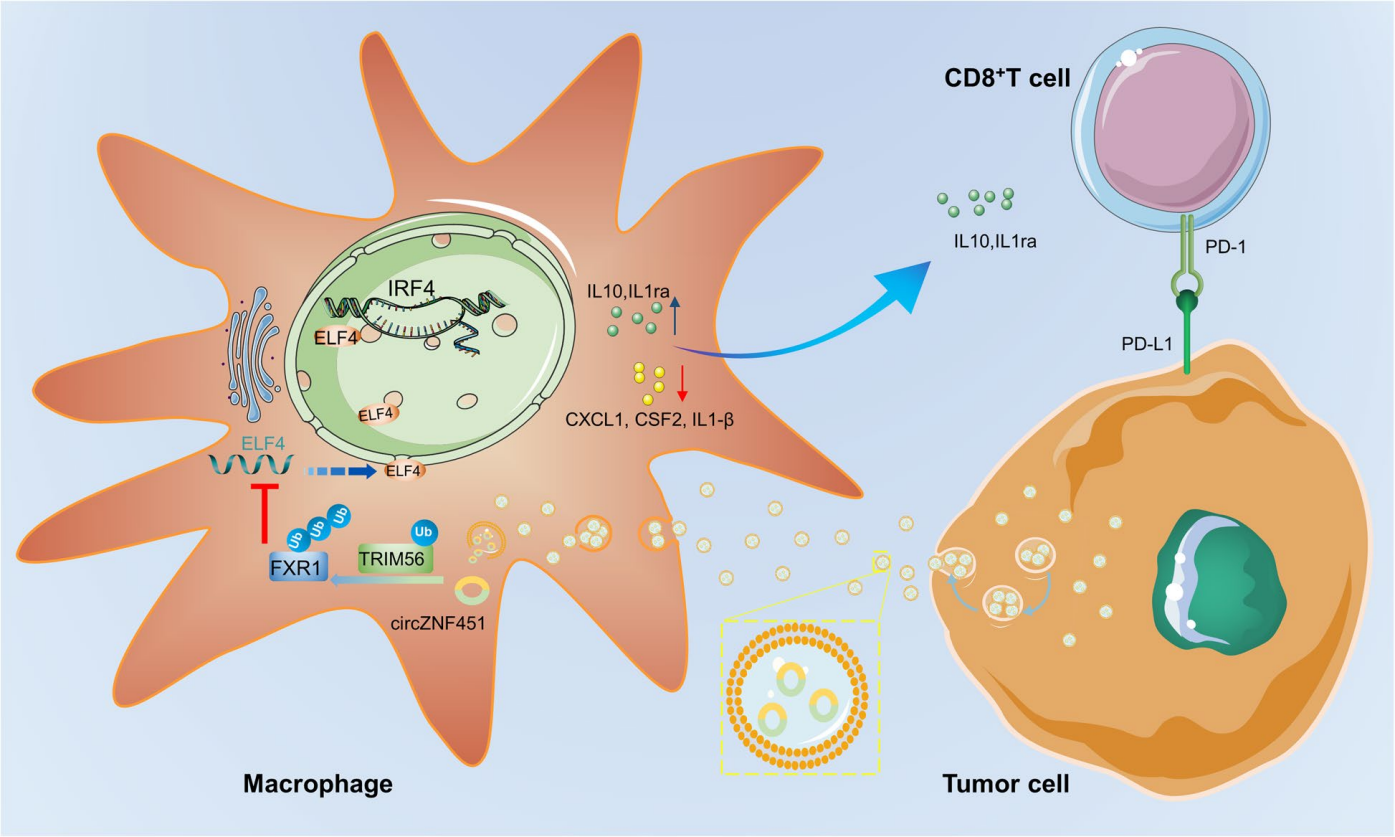
The diagram of this study. The LUAD derived exosomal circZNF451 could interact with FXR1 and TRIM56 and facilitate the ubiquitination of FXR1, which abrogates the inhibition on the downstream ELF4-IRF4 pathway and induces the pro-inflammatory phenotype of macrophages and the dysfunction of CD8^+^T cells

Here, we show that in LUAD, tumor-derived exosomal circZNF451 may elicit ubiquitination of FXR1 via the E3 ligase TRIM56 in macrophages. Degradation of FXR1 further reshapes the immune-suppressed TME via activation on the downstream ELF4–IRF4 pathway, which enhances the anti-inflammatory phenotype of macrophages and dysfunction of cytotoxic CD8^+^ T cells (Fig. 8) [22].

## Discussion

Exosomal circRNAs facilitate growth, metastasis, and drug resistance of cancers and can serve as a prognostic marker for patients. Cross-talk between tumors and immune cells via exosomal circRNAs also elicits reprogramming of TME immune status, which leads to immune evasion and resistance to immunotherapy [23]. Here, the circRNA sequencing was performed on the peripheral blood of six LUAD patients receiving PD1 blockade treatment. And to explore the enrichment profiles of circRNAs in exosomes, we identified significant enrichment of circZNF451 in the exosomes of PD patients compared with in PR patients, and expression was highly consistent with that observed in tumor tissues. Further analysis showed upregulation of circZNF451 in LUAD and highlighted its potential as a biomarker for poor LUAD prognosis. Moreover, circZNF451 interacts with FXR1 and TRIM56, forming the FXR1–circZNF451–TRIM56 complex in macrophages to facilitate degradation of FXR1 and further activation of the ELF4–IRF4 pathway, which induced M2 polarization of macrophages and the immune-suppressed TME. Thus, our study identified a new biomarker for the efficacy of immunotherapy in LUAD patients.

The biogenesis of circRNA is mediated by many factors including the splicing factors, specific enzymes, cis-acting elements and transcription factors [24], which collectively contributed to the differentially expression of circZNF451 and the discrepancy on the enrichment of circZNF451 in the exosomes among different cell lines.

CircRNAs have various mechanisms in cancer. Competing endogenous RNAs (ceRNAs) can bind miRNAs and further suppress their binding with mRNAs. CeRNAs can also modulate transcription, serve as a template for translation, and interact with proteins, including RBPs, to form circRNA–protein complexes [25]. RBPs are a group of proteins involved in transcription and translation, and circRNAs can elicit RBP degradation via the proteasome to regulate malignant properties and immune resistance [26, 27]. However, previous studies focused mainly on circRNA–RBP interactions in tumor cells, and investigation of this mechanism in the TME in LUAD was still limited. We identified that exosomal circZNF451 can act as a scaffold to induce FXR1 degradation via TRIM56-mediated ubiquitin activity in macrophages. Furthermore, circZNF451 is indispensable for the interaction between FXR1 and TRIM56 in that ubiquitination by TRIM56 relied on RNA-binding activity in its C terminus. Degradation of FXR1 further activated the transcription factor ELF4, induced the anti-inflammatory immune phenotype of macrophages, and reset the immune-suppressed TME, suggesting that this novel circRNA–RBP complex in macrophages may contribute to immunotherapy resistance in LUAD.

The TME, which changes during tumor growth, contains complicated cellular components, such as stromal cells, endothelial cells, and various immune subgroups. Interactions within these components reshape the malignant properties of tumor cells, and exosomes have critical roles in facilitating communication between tumor cells and these components [28]. Thus, exosomes contribute to modulation of the TME and represent a novel target for the enhancement of antitumor immunity in immune cells. Exosomal circRNAs in the TME regulate the immune response in diverse immune cells, including macrophages, lymphocytes, and NK cells, foster the immune-suppressed TME for the progression of cancers, and could also modulate drug resistance, including to chemotherapy, radiation, targeted therapy, and immunotherapy, via the miRNA–mRNA axis or interaction with proteins [10]. In this study, PD patients receiving PD1 blockade therapy had high enrichment of circZNF451 in exosomes. Exosomal circZNF451 could induce the anti-inflammatory phenotype of macrophages via interactions with FXR1 and foster the immunosuppressed TME to enhance resistance to immunotherapy. Thus, our study provides a potential target to modulate drug resistance in LUAD patients.

The transcription factor ELF4 can regulate the development and cytotoxic cytokine expression of NK and CD8^+^ T cells, the type I IFN response, activation of housekeeping genes in macrophages, and differentiation of CD4^+^ T cells to the T helper 17 cell subgroup, highlighting the importance of ELF4 in innate and adaptive immunity [29]. ELF4 is a mediator of anti-inflammatory responses and protects against mucosal disease in human inflammatory bowel disease [19]. We identified the impacts of ELF4 on macrophage inflammatory responses, which could enhance the anti-inflammatory immune phenotype and induce M2 polarization of macrophages. We also illustrated that activation of ELF4 is dependent on degradation of upstream FXR1 by the circZNF451–TRIM56 complex.

IRF4 mediates M2 polarization of macrophages, and activation of IRF4 via mTORC2 leads to metabolic reprogramming, which is indispensable for M2 activation [30]. Demethylation of H3K27 mediated by Jmjd3 is essential for activation of the expression of IRF4, resulting in M2 macrophage polarization [31]. Here, we analyzed ChIP data from a previously published study and demonstrated that ELF4 can bind to the promoter region of IRF4, which enhanced IRF4 transcription. We also found that conditional knockout of ELF4 in macrophages rescued the immunosuppressive TME and abrogated resistance to immunotherapy. Thus, our study provides a novel target that regulates the transcriptional activity of IRF4 in macrophages and a new strategy for enhancing immunotherapy efficacy in a subgroup of LUAD patients.

The in vivo results identified that conditional knockout of ELF4 in macrophages enhanced the efficacy of PD1 blockade in LUAD with the enhanced infiltration of CD8^+^ T cells and exhausted M2 macrophages. Although the PD1 monotherapy could not influence the infiltration of CD8^+^ T cells and M2 macrophages, the flow cytometry results suggested that the cytotoxic function of CD8^+^ T cells was partially improved with the increased secretion of IFN-γ and GZMB. In this study, we observed that M2 macrophages could impede the proliferation of CD8^+^T cells in vitro, which might account for this phenomenon.

This study also has some limitations. First, we mainly focused on the impactions and mechanisms of circZNF451 on the TME, the influence of circZNF451 on the biological functions of tumor cells was still absent. Secondly, this study uncovered the role of exosomal circZNF451 in the immunotherapy in preclinical models and only ten LUAD patients accepting the PD1 blockade were included for further validation. Thus, larger clinical samples with PD1 immunotherapy are necessary to further validate the role of exosomal circZNF451 in the resistance to immunotherapy. Finally, the commercial inhibitors on ELF4 or IRF4 are still unavailable, which limits further investigation on the dual-blockade strategy in advanced LUAD patients with high expression of circZNF451.

## Conclusion

We showed that circZNF451 is highly enriched in the exosomes of LUAD patients refractory to PD1 blockade. CircZNF451 could elicit the anti-inflammatory phenotype of macrophages via TRIM56-mediated ubiquitination of FXR1 and activation on the ELF4–IRF4 pathway, which reshapes the immunosuppressive TME in LUAD. Thus, circZNF451 may serve as a novel biomarker in predicting immunotherapy efficacy and a target to overcome resistance to PD1 blockade.

## Supplementary Information

**Additional file 1.** The mass spectromy data of the band at 70kDa of circZNF451 RNA pulldown in macrophages.

**Additional file 2.** The immune related genes that differentially expressed in macrophages after the overexpression of circZNF451.

**Additional file 3.** The clinical information of 4 patients in whom the exosmes were extracted from the sera before and after surgery.

**Additional file 4. **

**Additional file 5:** **Supplementary Figure 1.** **A** The enrichment of 4 most significantly changed circRNAs (FC > 4, *P* < 0.05) of the circRNA sequencing in the exosomes of 6 LUAD patients (3 PR and 3 PD) accepting the PD1 blockade was measured by qRT-PCR. **B** Correlation between exosomal circRNA-seq results of circZNF451 and the expression of circZNF451 in the tumor tissues of six LUAD patients was analyzed by Spearman correlation analysis. **C** The expression of circZNF451 in five LUAD cell lines (A549, H1299, Calu-3, H1975, and H1395) was detected by qRT-PCR. **D** The change on the expression of circZNF451 in nucleus and cytoplasm of H1395 and H1975 after the overexpression of circZNF451 was measured by qRT-PCR. **A** and **D** was analyzed by the two-tailed, unpaired Student’s *t* test. **C** was analyzed using one-way ANOVA test after adjusting for multiple comparisons. All experiments with statistical analysis have been repeated for at least three times.

**Additional file 6:** **Supplementary Figure 2.** **A** Infiltration of CD4^+^T, CD8^+^T, Foxp3^+^Treg, CD56^+^NK and CD11C^+^dendritic cells and CD86^+^ and CD163^+^ macrophages in the circZNF451^high^ and circZNF451^low^ group was presented in the violin plot. **B** The transfection efficiency of circZNF451 in LLC was measured by qRT-PCR. **C** The construction of A549-shcircZNF451, H1299-shcircZNF451, H1395-circZNF451 and H1975-circZNF451 cell lines was confirmed by qRT-PCR. **D** IFN-γ and GZMB levels in the supernatant of CD8^+^ T cells were measured via ELISA after coculturing with H1395-circZNF451 cells for 48 h. **E** M2 phenotype markers Arg1 and IL-10 in LPS stimulated macrophages were detected via qRT-PCR after coculturing with H1395-circZNF451 cells for 48 h. **F** CircZNF451 enrichment in macrophages cocultured with H1395-circZNF451 cells and GW4869 for 48 h was measured by qRT-PCR. **G** The M2 phenotype of LPS-stimulated macrophages cocultured with H1395-circZNF451 cells and GW4869 (20 μM) for 48 h was analyzed by flow cytometry. **H** The expression of M2 markers Arg1 and IL-10 in LPS-stimulated macrophages was measured by qRT-PCR after coculturing with H1395-circZNF451 cells and GW4869 (20 μM) for 48 h. **I** Concentrations of IFN-γ and GZMB in the supernatant of CD8^+^ T cells were measured by ELISA after stimulation by the supernatant from the LPS-stimulated macrophage/H1395-circZNF451. **J** After the stimulation by the supernatant from the LPS-stimulated macrophage/H1395-circZNF451 for 5 days, the proliferation of CD8^+^ T cells was detected by CFSE. **K** The M2 phenotype of LPS stimulated macrophages cultured with the A549-shcircZNF451 for 48h was analyzed by flow cytometry. **L** The enrichment of circZNF451 in the macrophages cultured with A549-shcircZNF451 for 48h was confirmed by qRT-PCR. **M** IFN-γ and GZMB levels in the supernatant of CD8^+^T cells were measured via ELISA after coculturing with the supernatant from LPS-stimulated macrophage/A549-shcircZNF451 cells for 48 h. **N** The proliferation of CD8^+^ T cells cultured by the supernatant from the LPS-stimulated macrophage/A549-shcircZNF451 cells for 5 days was measured by CFSE. **A-E**, I and J were analyzed by the two-tailed, unpaired Student’s *t* test. **F-H** and **K-N** was analyzed using one-way ANOVA test after adjusting for multiple comparisons. All experiments with statistical analysis have been repeated for at least three times.

**Additional file 7:** **Supplementary Figure 3.** **A** Silver staining of the complexes precipitated by the biotinylated circZNF451/NC probe. The red arrow marks the specific band of the circZNF451 probe. **B** The overlap of the mass spectrometry data, the predicted candidates for circZNF451 in circInteractome and the most detected binding motifs for circZNF451 in catRAPID database. **C** After coculturing with H1395/H1975-shcircZNF451 and GW4869 (20μM), FXR1 expression in macrophages was detected via western blotting. **D** and **E** The FXR1 binding motif (**D**) and the putative and mutant binding sites in circZNF451 (**E**). All experiments with statistical analysis have been repeated for at least three times

**Additional file 8:** **Supplementary Figure 4.** **A** After coculturing with A549 for 48h, RNA pull down was performed in macrophages transfected with TRIM56^mutant^ to confirm the interaction between circZNF451 and TRIM56^mutant^. **B** The co-IP assay was used to verify the interaction between FXR1 and TRIM56 in macrophage after coculturing with A549/H1299-shcircZNF451 cells for 48h. **C** The M2 phenotype markers Arg1 and IL-10 were detected by qRT-PCR in LPS stimulated macrophage-siFXR1 cells stimulated by LPS. **D** The M2 phenotype of LPS stimulated macrophage-TRIM56^mutant^ cells was detected by flow cytometry after coculturing with A549 cells. **E** The M2 phenotype markers Arg1 and IL-10 were detected by qRT-PCR in LPS stimulated macrophage- TRIM56^mutant^ cells after coculturing with A549 cells. **F** After the transfection of the vectors containing the 3’UTR of ELF4 and siFXR1 in macrophages, the relative luciferase activities were measured. Data was analyzed by one-way ANOVA test after adjusting for multiple comparisons. **C-D** were analyzed by Student’s *t* test was applied. All experiments with statistical analysis have been repeated for at least three times.

**Additional file 9:** **Supplementary Figure 5.** **A** Expression of ELF4 in macrophages cocultured with H1395-circZNF451 cells and the macrophages with silence of FXR1 was detected by western blotting. **B** The expression of ELF4 in macrophages cocultured with H1395-circZNF451 cells and the macrophages with silence of FXR1 was detected by qRT-PCR. **C** GM-CSF, CXCL1, IL-1β, IL-1Ra, and IL-10 levels in the supernatant of LPS stimulated macrophage-siELF4 cells were measured by ELISA. **D** IRF4 expression in LPS stimulated macrophage-siELF4 cells was measured by qRT-PCR. **E** IRF4 expression in LPS stimulated macrophages cocultured with LUAD cell lines with silenced (A549 and H1299) or overexpressed (H1395 and H1975) circZNF451 and the administration of GW4869 (20μM) was measured by qRT-PCR. **F** GM-CSF, CXCL1, IL-1β, IL-1Ra, and IL-10 levels in the supernatant of LPS stimulated macrophage-siIRF4 cells were measured by ELISA. **B-E** used one-way ANOVA test after adjusting for multiple comparisons. F was compared by two-tailed, unpaired Student’s *t* test. All experiments with statistical analysis have been repeated for at least three times.

**Additional file 10:** **Supplementary Figure 6.** **A** Image of subcutaneous tumors of four groups implanted with LLC-circZNF451: WT + PBS, WT + PD1 blockade, ELF4 KO + PBS, and ELF4 KO + PD1 (*n* = 5 for each group) and the statistical analysis on the tumor growth of the four groups. The conditional knockout of ELF4 is accomplished with C57BL/6J-Lyz2^CreERT2^ and C57BL/6J-ELF4^em1(^^flox^^)^^Smoc^ mice; KO, knockout. **B** TIM3 expression and GZMB secretion in CD8^+^ T cells in LLC-circZNF451 cell WT + PBS, WT + PD1 blockade, ELF4 KO + PBS, and ELF4 KO + PD1 groups (*n* = 5 per group) were analyzed by flow cytometry. All experiments with statistical analysis have been repeated for at least three times. One-way ANOVA test after adjusting for multiple comparisons was performed.

**Additional file 11. **Supplementary methods.

**Additional file 12:** **Supplementary Table 1.** The clinical information of six LUAD patients accepted the PD1 blockade and underwent circRNA-seq of the peripheral blood. **Supplementary Table 2.** The clinical characteristics of 113 LUAD patients with different expression of circZNF451. **Supplementary Table 3.** Target sequences of shRNA and siRNA used in this study. **Supplementary Table 4.** The antibodies, ELISA kits and drugs used in this study. **Supplementary Table 5.** The primers and probes used in this study. **Supplementary Table 6.** The validation set of 10 LUAD patients accepting the PD1 blockade.

## Data Availability

The datasets used and/or analysed during the current study are available from the corresponding author on reasonable request.

## Abbreviations

TME: Tumor microenvironment
LUAD: Lung adenocarcinoma
circRNA: Circular RNA
NK cell: Natural killer cell
PD1: Programmed death 1
Treg: Regulatory T cell
RBP: RNA-binding protein
TMA: Tumor microarray
FC: Fold change
KEGG: Kyoto Encyclopedia of Genes and Genomes
FISH: Fluorescence in situ hybridization
IRF: Interferon regulatory factor
IFN-γ: Interferon-γ
GZMB: Granzyme B
qRT-PCR: Quantitative real-time PCR
bp: Base pairs
WT: Wild-type
SDS: Sodium dodecyl sulfate
RIP: RNA immunoprecipitation
miRNA: MicroRNA
PD: Progressive disease
PR: Partial remission
GO: Gene Ontology
PBS: Phosphate-buffered saline
IL: Interleukin
IL-1Ra: IL-1 receptor antagonist
GM-CSF: Granulocyte–macrophage colony-stimulating factor
ELISA: Enzyme-linked immunosorbent assay
ceRNA: Competing endogenous RNA
PMA: Phorbol myristate acetate
IHC: Immunohistochemistry
co-IP: Coimmunoprecipitation
ChIP: Chromatin immunoprecipitation
TAMs: Tumor-associated macrophages
