# Supplementary material for: Exosomal circZNF451 restrains anti-PD1 treatment in lung adenocarcinoma via polarizing macrophages by complexing with TRIM56 and FXR1
Source: J Exp Clin Cancer Res. 2022 Oct 8;41:295. doi: 10.1186/s13046-022-02505-z (PMC9547453; doi:10.1186/s13046-022-02505-z)
Supplement: Supplementary file 4 — Additional file 4. [file 13046_2022_2505_MOESM4_ESM.pdf]

Figure 1C

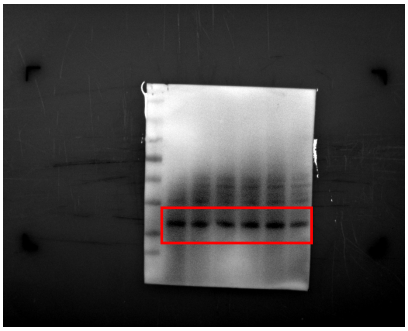

Exosome-CD63

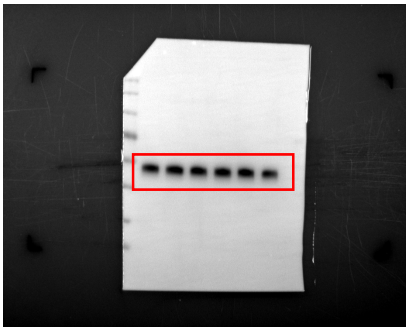

Exosome-TSG101

Figure 3B

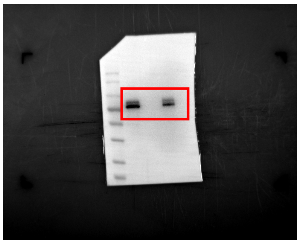

circZNF451 pulldown FXR1

Figure 3C

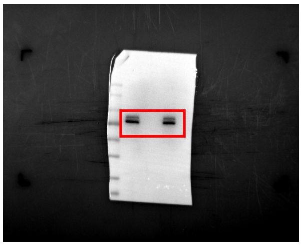

circZNF451 RIP FXR1

Figure 3H

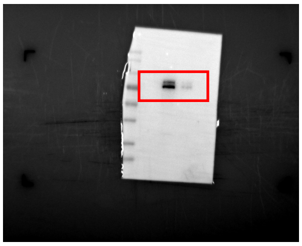

Macrophage FXR1 pulldown

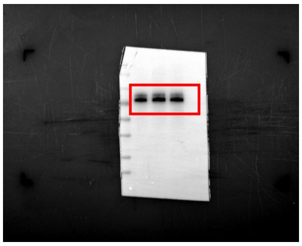

Macrophage FXR1 Input

Figure 3E

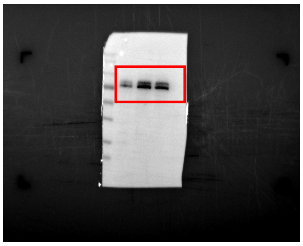

macrophage^{A549} FXR1

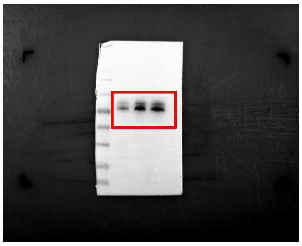

macrophage^{H1299} FXR1

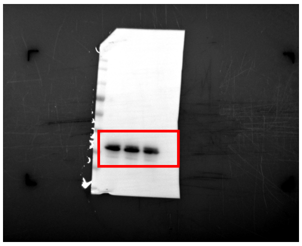

macrophage^{A549} GAPDH

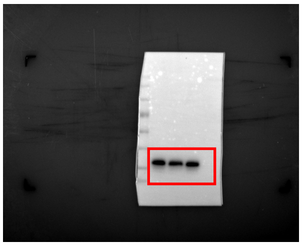

macrophage^{H1299} GAPDH

Figure 3I

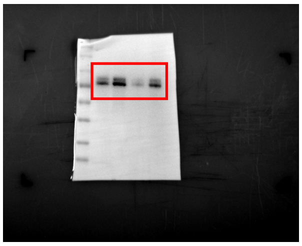

macrophage^{H1395} FXR1

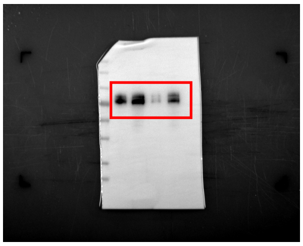

macrophage^{H1975} FXR1

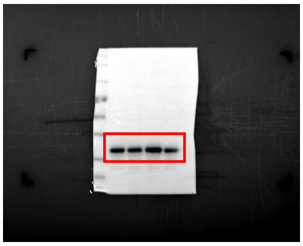

macrophage^{H1395} GAPDH

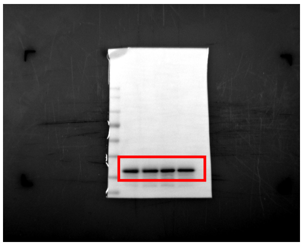

macrophage^{H1975} GAPDH

Figure 3J

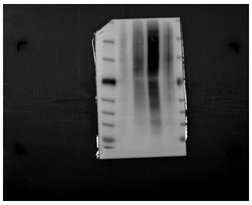

macrophage^{H1395} circZNF451 FXR1 Ub

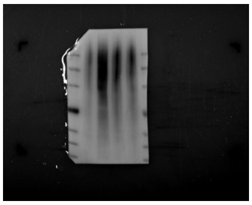

macrophage^{A549} shcircZNF451 FXR1 Ub

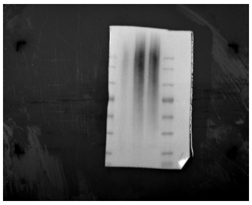

macrophage^{H1395} GW4869 FXR1 Ub

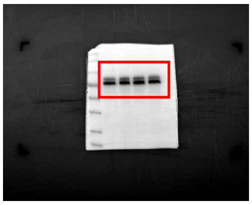

macrophage^{H1395} circZNF451 FXR1 Input

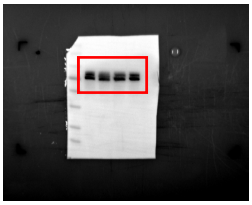

macrophage^{A549} shcircZNF451 FXR1 Input

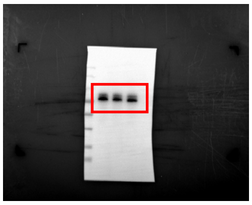

macrophage^{H1395} GW4869 FXR1 Input

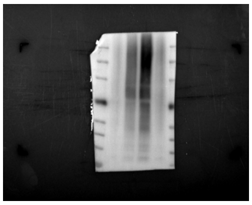

macrophage^{H1975} circZNF451 FXR1 Ub

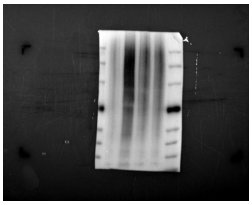

macrophage^{H1299} shcircZNF451 FXR1 Ub

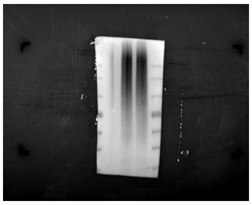

macrophage^{H1975} GW4869 FXR1 Ub

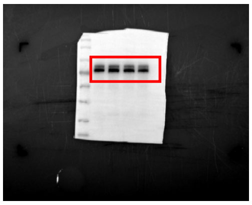

macrophage^{H1975} circZNF451 FXR1 Input

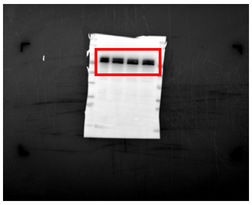

macrophage^{H1299} shcircZNF451 FXR1 Input

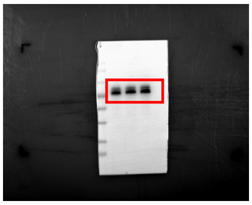

macrophage^{H1975} GW4869 FXR1 Input

Figure 4A

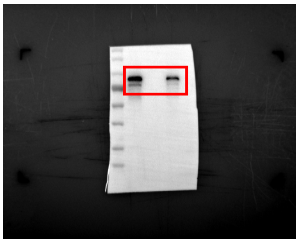

circZNF451 pulldown TRIM56

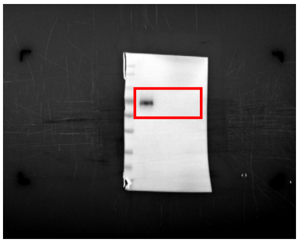

circZNF451 pulldown TRIM25

Figure 4B

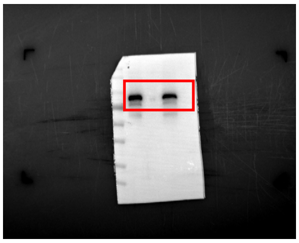

circZNF451 RIP TRIM56

Figure 4C

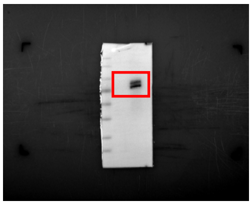

FXR1 IP

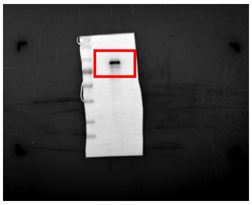

TRIM56 IP

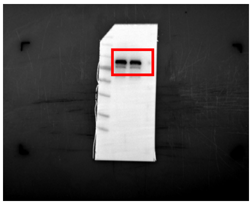

TRIM56 Input

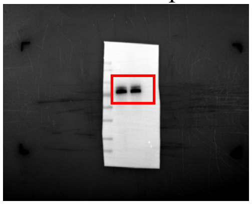

FXR1 Input

Figure 4D

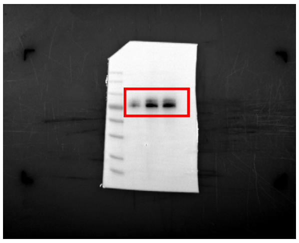

macrophage^{H1395} siTRIM56 FXR1

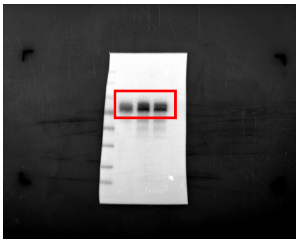

macrophage^{H1975} siTRIM56 FXR1

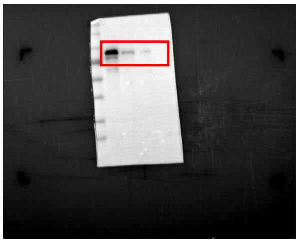

macrophage^{H1395} siTRIM56 TRIM56

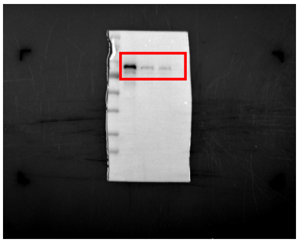

macrophage^{H1975} siTRIM56 TRIM56

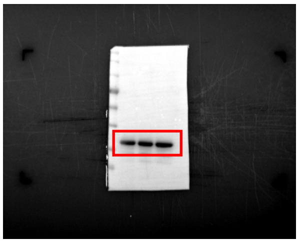

macrophage^{H1395} siTRIM56 GAPDH

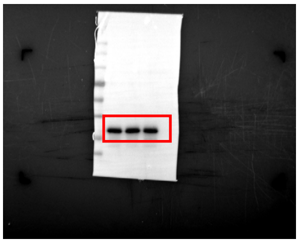

macrophage^{H1975} siTRIM56 GAPDH

Figure 4E

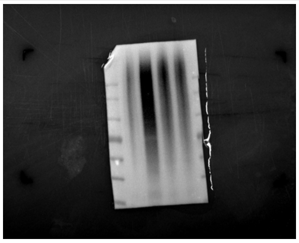

macrophage^{H1395} siTRIM56 FXR1 Ub

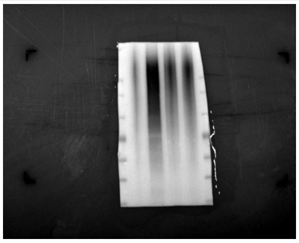

macrophage^{H1975} siTRIM56 FXR1 Ub

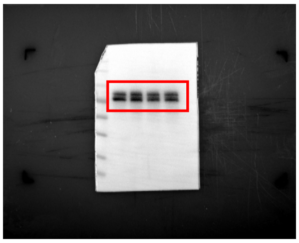

macrophage^{H1395} siTRIM56 FXR1 Input

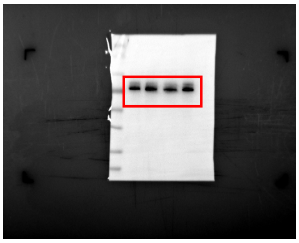

macrophage^{H1975} siTRIM56 FXR1 Input

Figure 4F

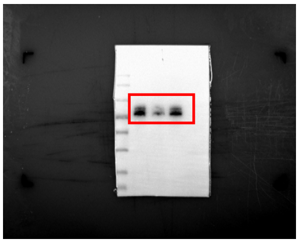

macrophage^{A549} TRIM56^{mutant} FXR1

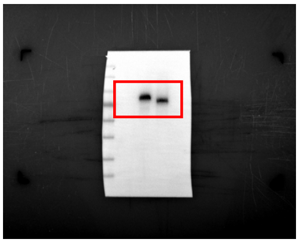

macrophage^{A549} TRIM56^{mutant} Flag

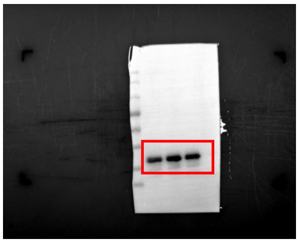

macrophage^{A549} TRIM56^{mutant} GAPDH

Figure 4G

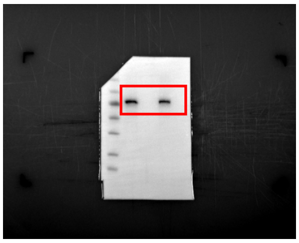

circZNF451 RIP TRIM56 mutant

Figure 4H

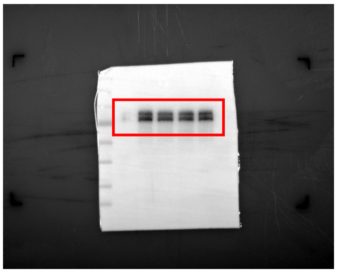

macrophage^{H1395} circZNF451^{mutant} FXR1 IP

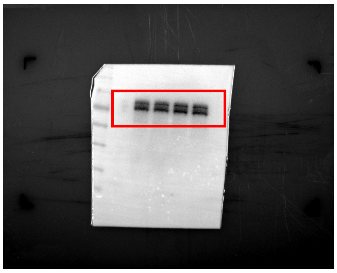

macrophage^{H1975} circZNF451^{mutant} FXR1 IP

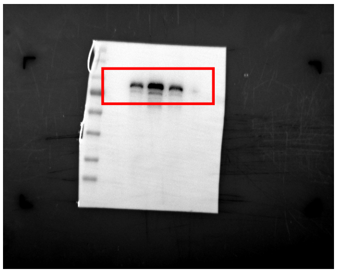

macrophage^{H1395} circZNF451^{mutant} TRIM56 IP

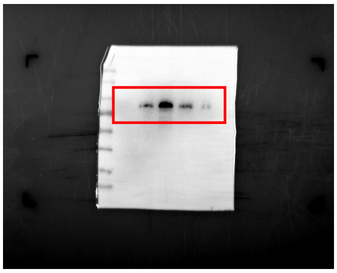

macrophage^{H1975} circZNF451^{mutant} TRIM56 IP

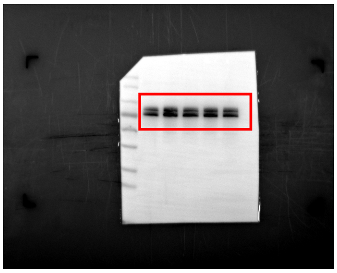

macrophage^{H1395} circZNF451^{mutant} FXR1 Input

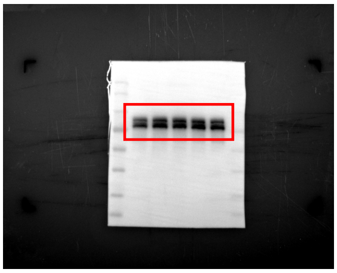

macrophage^{H1975} circZNF451^{mutant} FXR1 Input

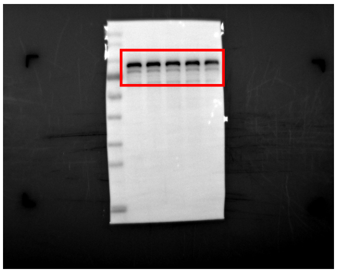

macrophage^{H1395} circZNF451^{mutant} TRIM56 Input

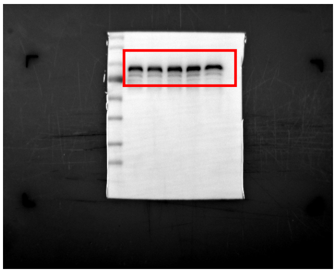

macrophage^{H1975} circZNF451^{mutant} TRIM56 Input

Figure 5A

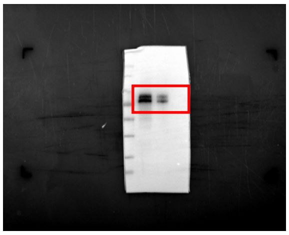

macrophage-siFXR1 FXR1

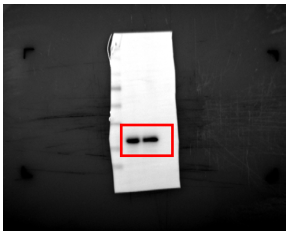

macrophage-siFXR1 GAPDH

Figure 6E

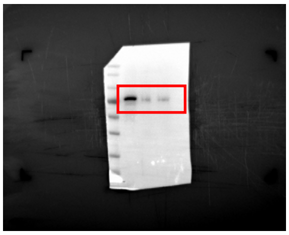

macrophage-siELF4 ELF4

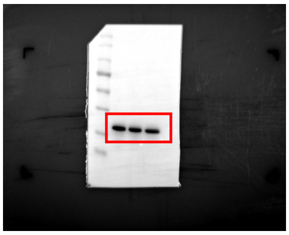

macrophage-siELF4 GAPDH

Figure 6K

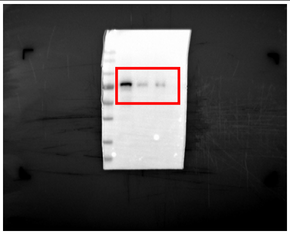

macrophage-siELF4 ELF4

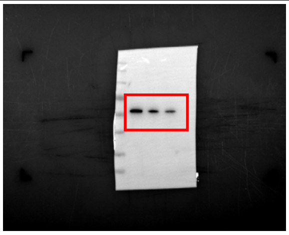

macrophage-siELF4 IRF4

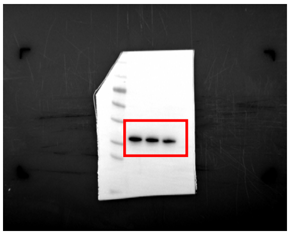

macrophage-siELF4 GAPDH

Figure 6L

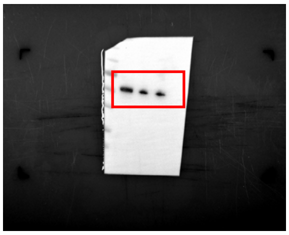

macrophage^{A549}shcircZNF451 IRF4

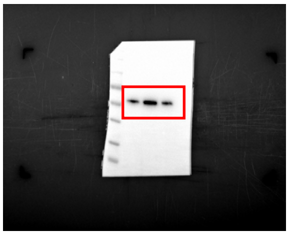

macrophage^{H1395} circZNF451 IRF4

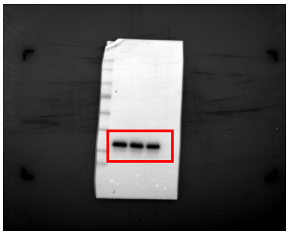

macrophage^{A549}shcircZNF451 GAPDH

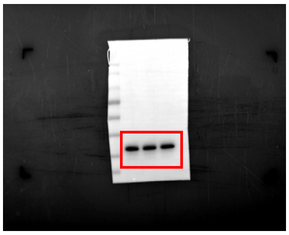

macrophage^{H1395} circZNF451 GAPDH

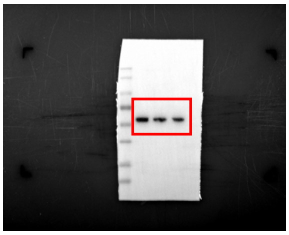

macrophage^{H1299}shcircZNF451 IRF4

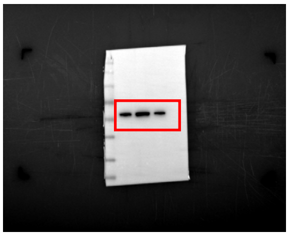

macrophage^{H1975} circZNF451 IRF4

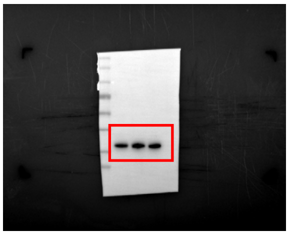

macrophage^{H1299}shcircZNF451 GAPDH

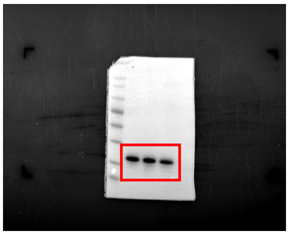

macrophage^{H1975} circZNF451 GAPDH

Supplementary Figure 3C

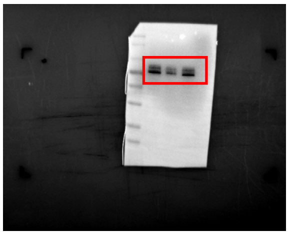

macrophage^{H1395} circZNF451 FXR1

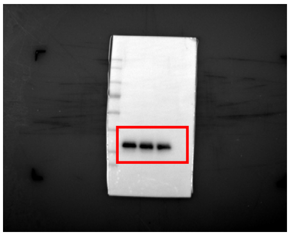

macrophage^{H1395} circZNF451 GAPDH

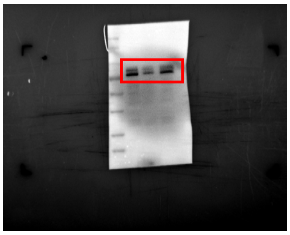

macrophage^{H1975} circZNF451 FXR1

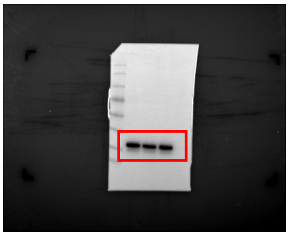

macrophage^{H1975} circZNF451 GAPDH

Supplementary Figure 4A

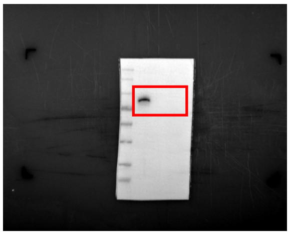

circZNF451 pulldown TRIM56 IP

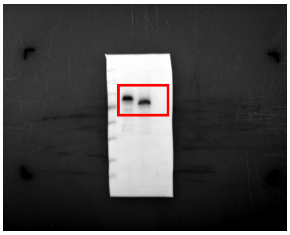

circZNF451 pulldown TRIM56 Input

Supplementary Figure 4B

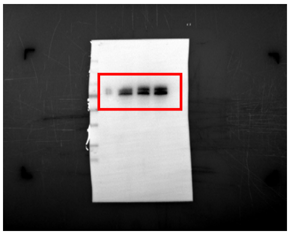

macrophage^{A549}shcircZNF451 FXR1 IB

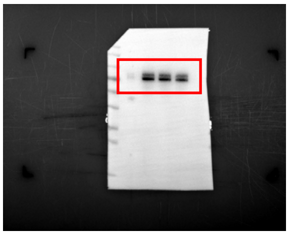

macrophage^{H1299}shcircZNF451 FXR1 IB

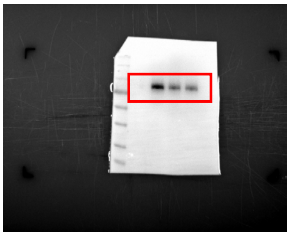

macrophage^{A549}shcircZNF451 TRIM56 IB

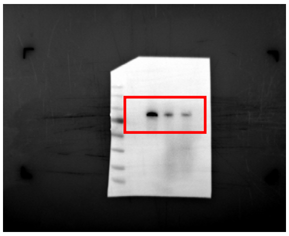

macrophage^{H1299}shcircZNF451 TRIM56 IB

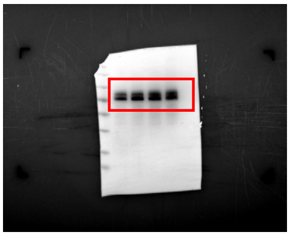

macrophage^{A549}shcircZNF451 FXR1 Input

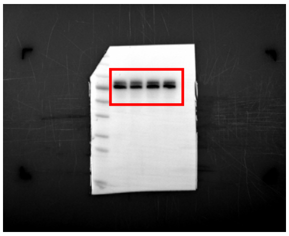

macrophage^{H1299}shcircZNF451 FXR1 Input

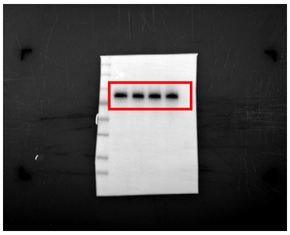

macrophage^{A549}shcircZNF451 TRIM56 Input

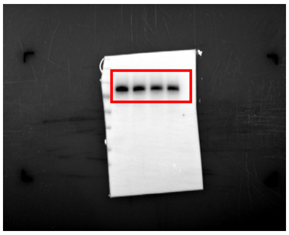

macrophage^{H1299}shcircZNF451 TRIM56 Input

Supplementary Figure 5A

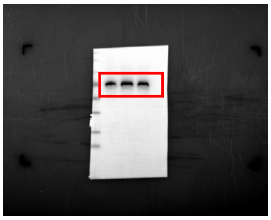

macrophage^{H1395} circZNF451 ELF4

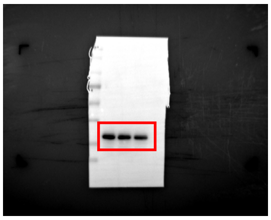

macrophage^{H1395} circZNF451 GAPDH
