# Supplementary figures and images for: Exosomal circZNF451 restrains anti-PD1 treatment in lung adenocarcinoma via polarizing macrophages by complexing with TRIM56 and FXR1
Source: J Exp Clin Cancer Res. 2022 Oct 8;41:295. doi: 10.1186/s13046-022-02505-z (PMC9547453; doi:10.1186/s13046-022-02505-z)

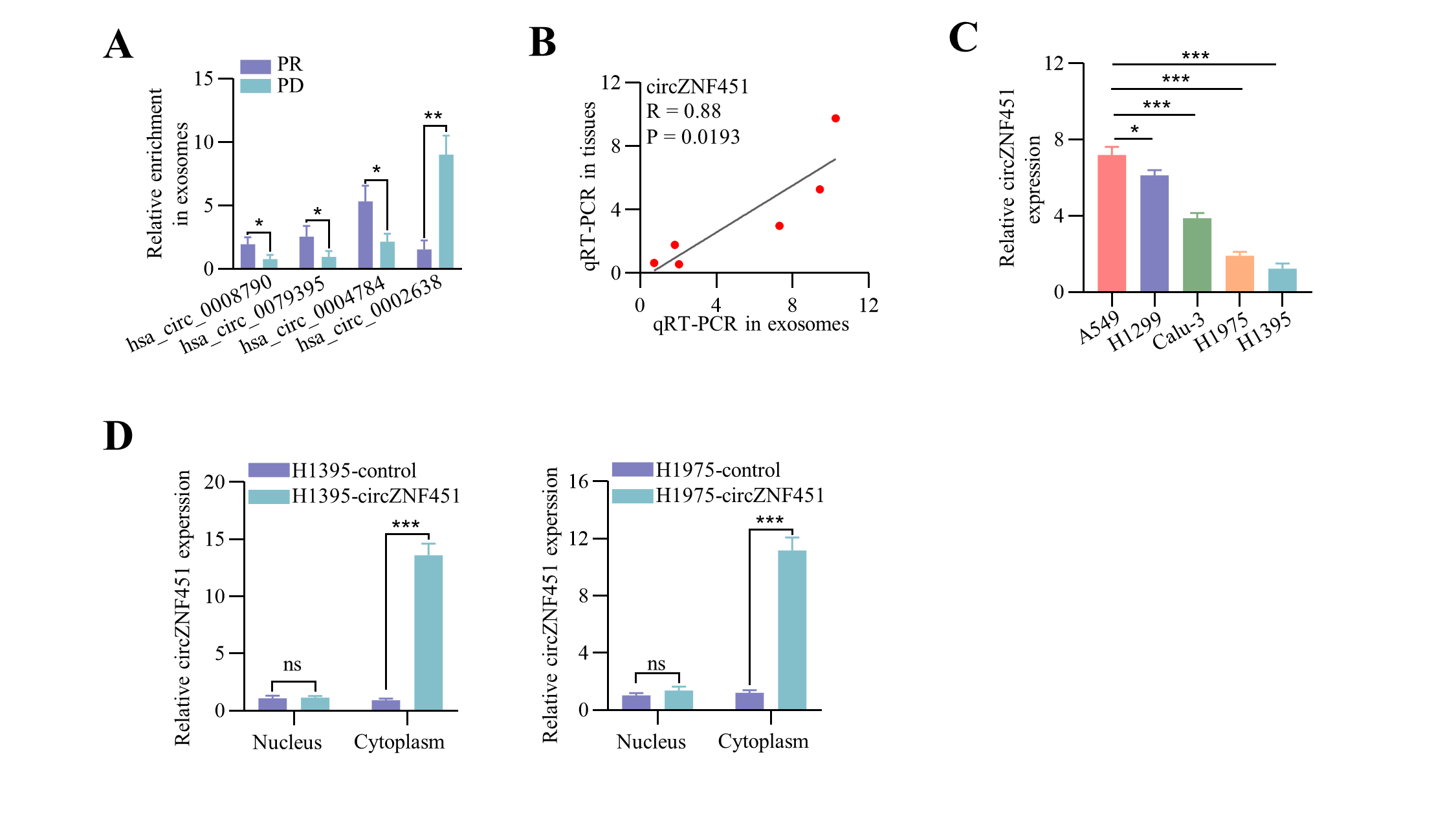

Supplement: Supplementary file 5 — Additional file 5: Supplementary Figure 1. A The enrichment of 4 most significantly changed circRNAs (FC > 4, P < 0.05) of the circRNA sequencing in the exosomes of 6 LUAD patients (3 PR and 3 PD) accepting the PD1 blockade was measured by qRT-PCR. B Correlation between exosomal circRNA-seq results of circZNF451 and the expression of circZNF451 in the tumor tissues of six LUAD patients was analyzed by Spearman correlation analysis. C The expression of circZNF451 in five LUAD cell lines (A549, H1299, Calu-3, H1975, and H1395) was detected by qRT-PCR. D The change on the expression of circZNF451 in nucleus and cytoplasm of H1395 and H1975 after the overexpression of circZNF451 was measured by qRT-PCR. A and D was analyzed by the two-tailed, unpaired Student’s t test. C was analyzed using one-way ANOVA test after adjusting for multiple comparisons. All experiments with statistical analysis have been repeated for at least three times. [file 13046_2022_2505_MOESM5_ESM.tif]

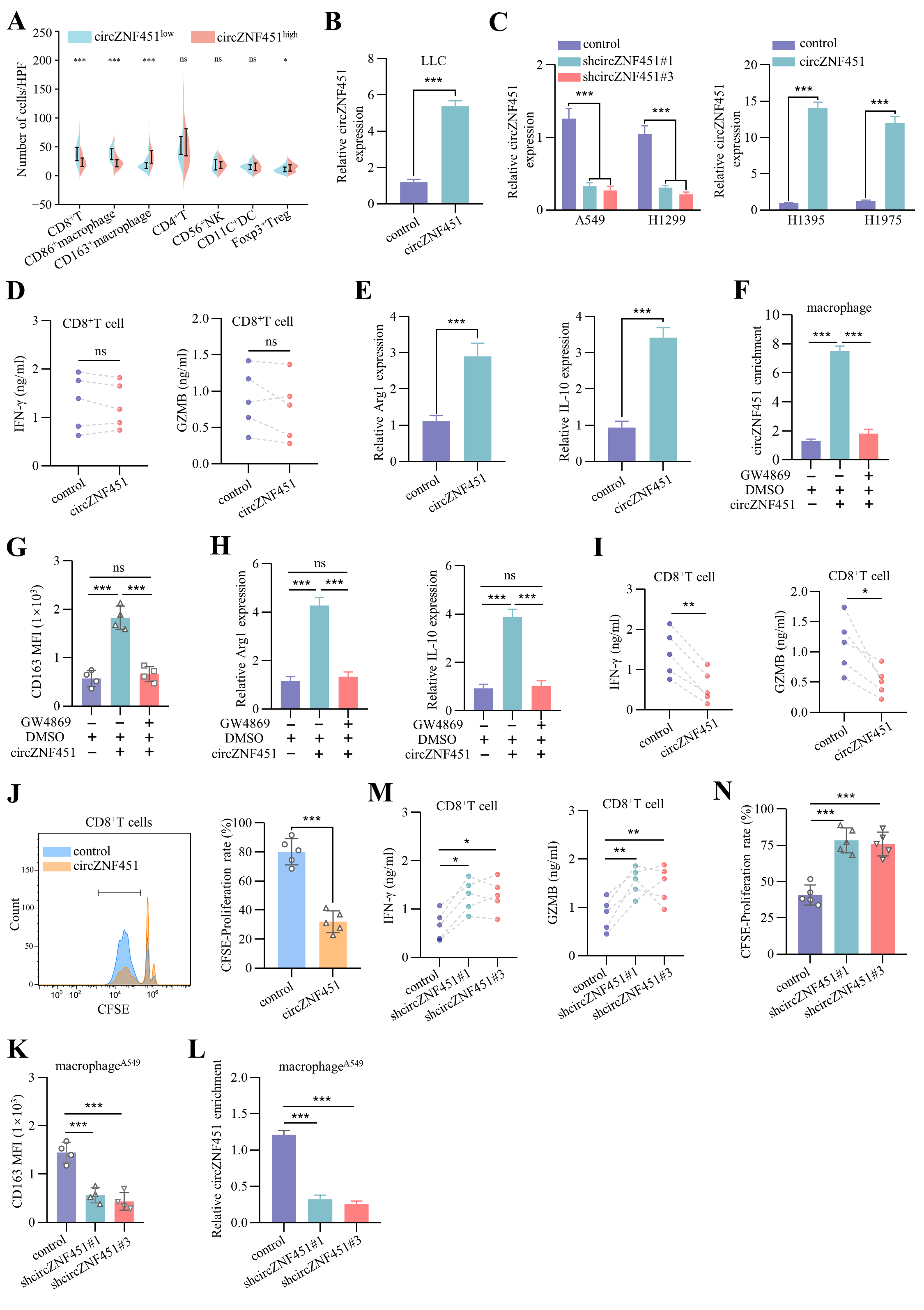

Supplement: Supplementary file 6 — Additional file 6: Supplementary Figure 2. A Infiltration of CD4+T, CD8+T, Foxp3+Treg, CD56+NK and CD11C+dendritic cells and CD86+ and CD163+ macrophages in the circZNF451high and circZNF451low group was presented in the violin plot. B The transfection efficiency of circZNF451 in LLC was measured by qRT-PCR. C The construction of A549-shcircZNF451, H1299-shcircZNF451, H1395-circZNF451 and H1975-circZNF451 cell lines was confirmed by qRT-PCR. D IFN-γ and GZMB levels in the supernatant of CD8+ T cells were measured via ELISA after coculturing with H1395-circZNF451 cells for 48 h. E M2 phenotype markers Arg1 and IL-10 in LPS stimulated macrophages were detected via qRT-PCR after coculturing with H1395-circZNF451 cells for 48 h. F CircZNF451 enrichment in macrophages cocultured with H1395-circZNF451 cells and GW4869 for 48 h was measured by qRT-PCR. G The M2 phenotype of LPS-stimulated macrophages cocultured with H1395-circZNF451 cells and GW4869 (20 μM) for 48 h was analyzed by flow cytometry. H The expression of M2 markers Arg1 and IL-10 in LPS-stimulated macrophages was measured by qRT-PCR after coculturing with H1395-circZNF451 cells and GW4869 (20 μM) for 48 h. I Concentrations of IFN-γ and GZMB in the supernatant of CD8+ T cells were measured by ELISA after stimulation by the supernatant from the LPS-stimulated macrophage/H1395-circZNF451. J After the stimulation by the supernatant from the LPS-stimulated macrophage/H1395-circZNF451 for 5 days, the proliferation of CD8+ T cells was detected by CFSE. K The M2 phenotype of LPS stimulated macrophages cultured with the A549-shcircZNF451 for 48h was analyzed by flow cytometry. L The enrichment of circZNF451 in the macrophages cultured with A549-shcircZNF451 for 48h was confirmed by qRT-PCR. M IFN-γ and GZMB levels in the supernatant of CD8+T cells were measured via ELISA after coculturing with the supernatant from LPS-stimulated macrophage/A549-shcircZNF451 cells for 48 h. N The proliferation of CD8+ T cells cultured b [file 13046_2022_2505_MOESM6_ESM.tif]

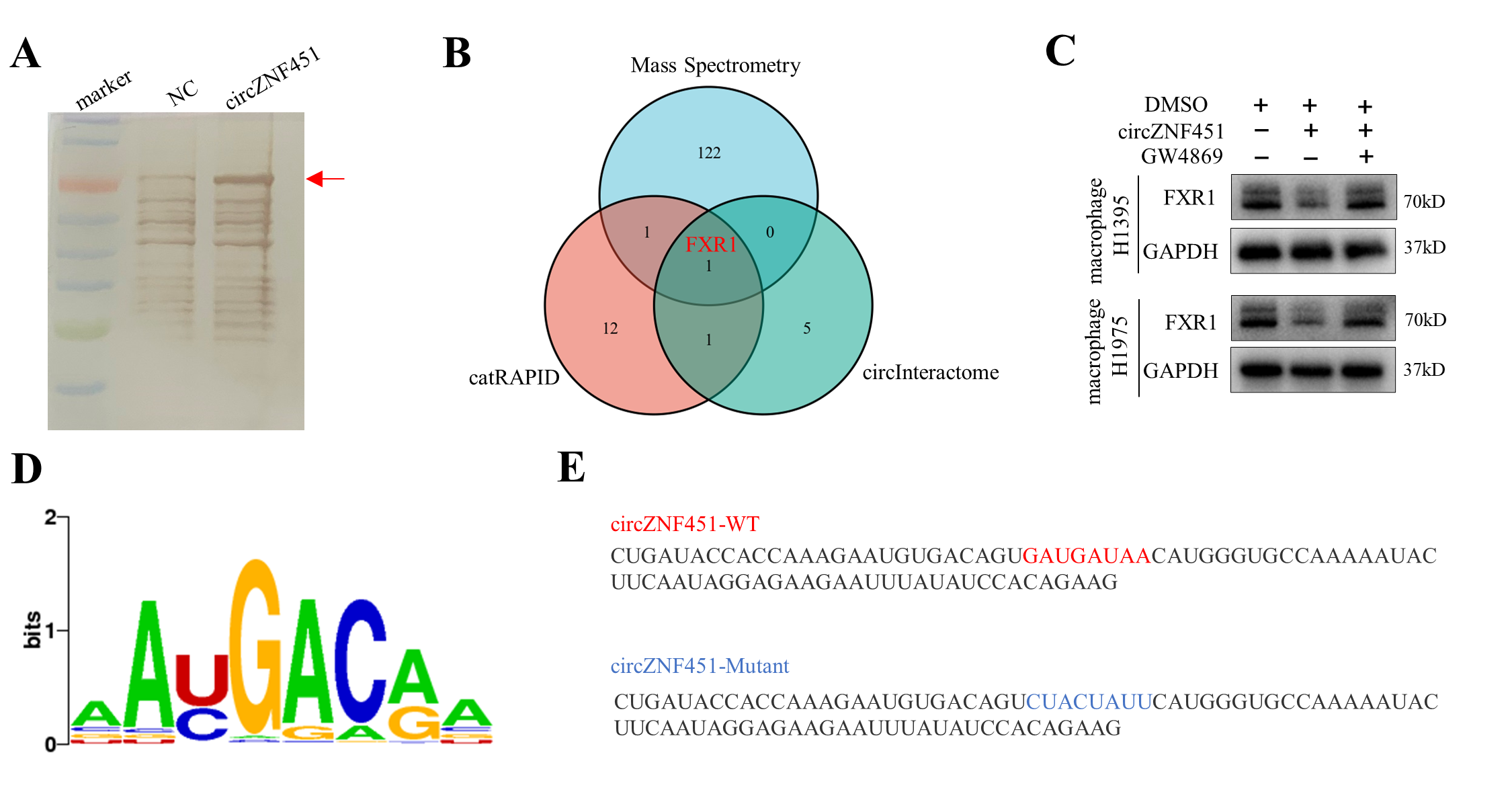

Supplement: Supplementary file 7 — Additional file 7: Supplementary Figure 3. A Silver staining of the complexes precipitated by the biotinylated circZNF451/NC probe. The red arrow marks the specific band of the circZNF451 probe. B The overlap of the mass spectrometry data, the predicted candidates for circZNF451 in circInteractome and the most detected binding motifs for circZNF451 in catRAPID database. C After coculturing with H1395/H1975-shcircZNF451 and GW4869 (20μM), FXR1 expression in macrophages was detected via western blotting. D and E The FXR1 binding motif (D) and the putative and mutant binding sites in circZNF451 (E). All experiments with statistical analysis have been repeated for at least three times [file 13046_2022_2505_MOESM7_ESM.tif]

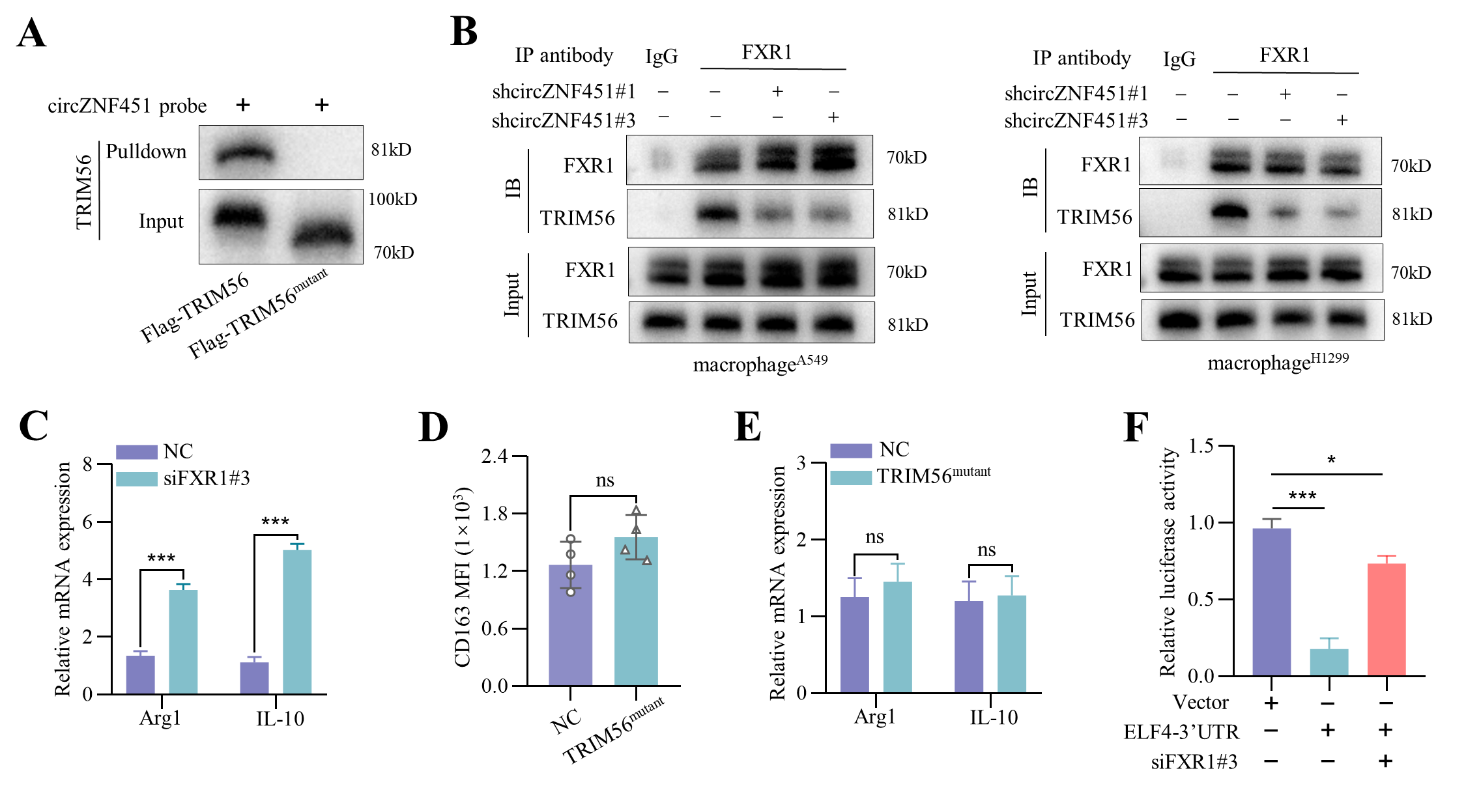

Supplement: Supplementary file 8 — Additional file 8: Supplementary Figure 4. A After coculturing with A549 for 48h, RNA pull down was performed in macrophages transfected with TRIM56mutant to confirm the interaction between circZNF451 and TRIM56mutant. B The co-IP assay was used to verify the interaction between FXR1 and TRIM56 in macrophage after coculturing with A549/H1299-shcircZNF451 cells for 48h. C The M2 phenotype markers Arg1 and IL-10 were detected by qRT-PCR in LPS stimulated macrophage-siFXR1 cells stimulated by LPS. D The M2 phenotype of LPS stimulated macrophage-TRIM56mutant cells was detected by flow cytometry after coculturing with A549 cells. E The M2 phenotype markers Arg1 and IL-10 were detected by qRT-PCR in LPS stimulated macrophage- TRIM56mutant cells after coculturing with A549 cells. F After the transfection of the vectors containing the 3’UTR of ELF4 and siFXR1 in macrophages, the relative luciferase activities were measured. Data was analyzed by one-way ANOVA test after adjusting for multiple comparisons. C-D were analyzed by Student’s t test was applied. All experiments with statistical analysis have been repeated for at least three times. [file 13046_2022_2505_MOESM8_ESM.tif]

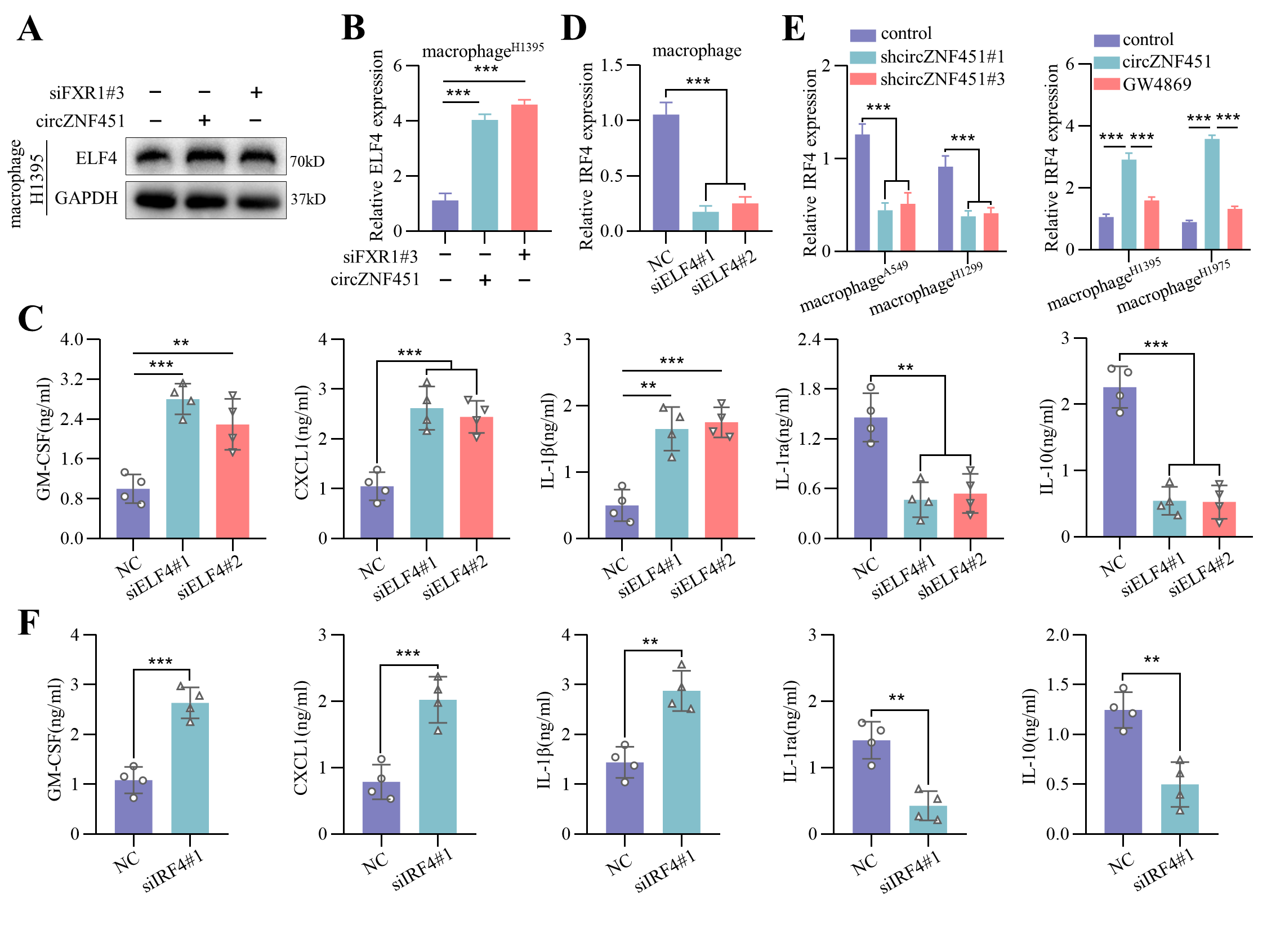

Supplement: Supplementary file 9 — Additional file 9: Supplementary Figure 5. A Expression of ELF4 in macrophages cocultured with H1395-circZNF451 cells and the macrophages with silence of FXR1 was detected by western blotting. B The expression of ELF4 in macrophages cocultured with H1395-circZNF451 cells and the macrophages with silence of FXR1 was detected by qRT-PCR. C GM-CSF, CXCL1, IL-1β, IL-1Ra, and IL-10 levels in the supernatant of LPS stimulated macrophage-siELF4 cells were measured by ELISA. D IRF4 expression in LPS stimulated macrophage-siELF4 cells was measured by qRT-PCR. E IRF4 expression in LPS stimulated macrophages cocultured with LUAD cell lines with silenced (A549 and H1299) or overexpressed (H1395 and H1975) circZNF451 and the administration of GW4869 (20μM) was measured by qRT-PCR. F GM-CSF, CXCL1, IL-1β, IL-1Ra, and IL-10 levels in the supernatant of LPS stimulated macrophage-siIRF4 cells were measured by ELISA. B-E used one-way ANOVA test after adjusting for multiple comparisons. F was compared by two-tailed, unpaired Student’s t test. All experiments with statistical analysis have been repeated for at least three times. [file 13046_2022_2505_MOESM9_ESM.tif]

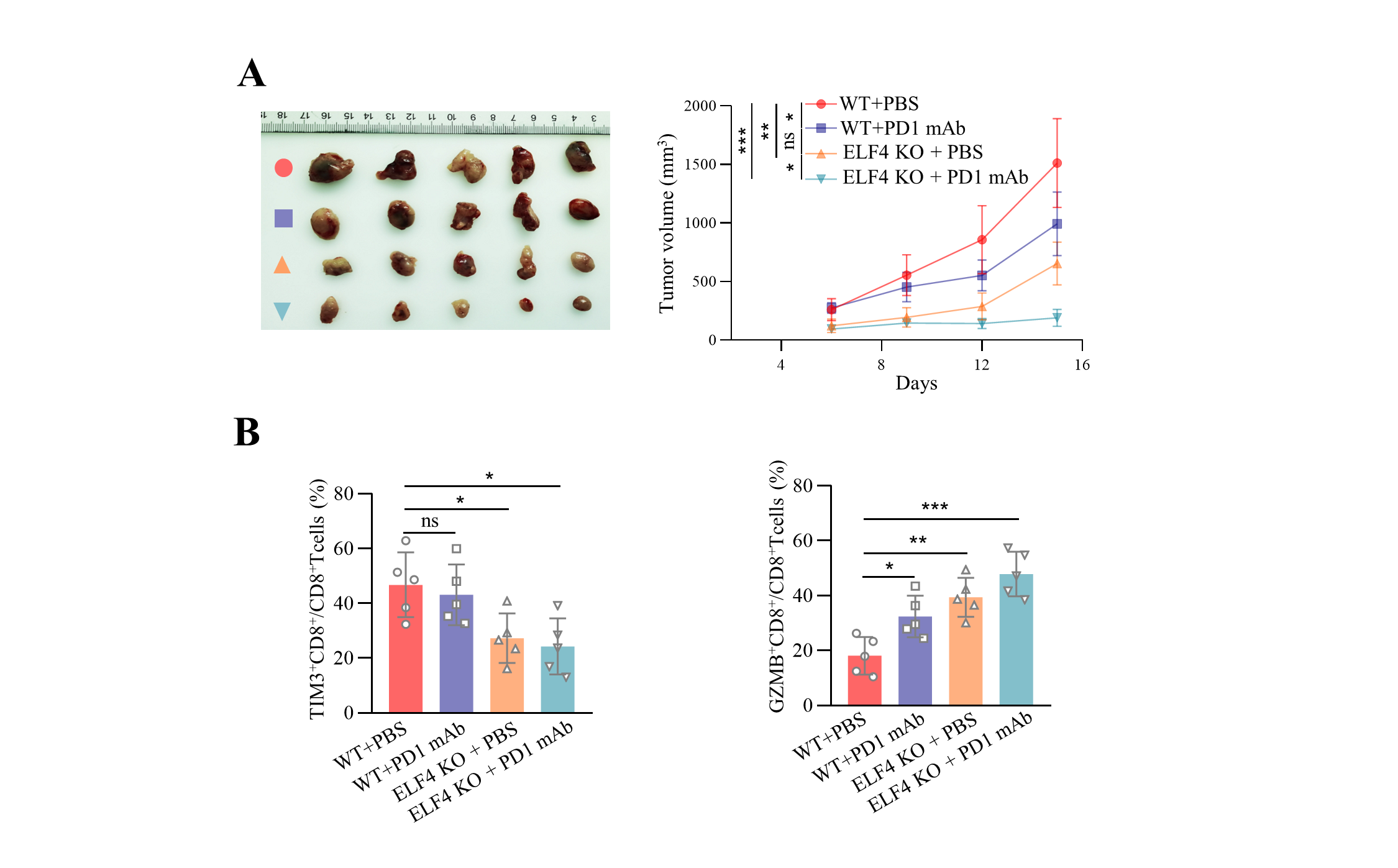

Supplement: Supplementary file 10 — Additional file 10: Supplementary Figure 6. A Image of subcutaneous tumors of four groups implanted with LLC-circZNF451: WT + PBS, WT + PD1 blockade, ELF4 KO + PBS, and ELF4 KO + PD1 (n = 5 for each group) and the statistical analysis on the tumor growth of the four groups. The conditional knockout of ELF4 is accomplished with C57BL/6J-Lyz2CreERT2 and C57BL/6J-ELF4em1(flox)Smoc mice; KO, knockout. B TIM3 expression and GZMB secretion in CD8+ T cells in LLC-circZNF451 cell WT + PBS, WT + PD1 blockade, ELF4 KO + PBS, and ELF4 KO + PD1 groups (n = 5 per group) were analyzed by flow cytometry. All experiments with statistical analysis have been repeated for at least three times. One-way ANOVA test after adjusting for multiple comparisons was performed. [file 13046_2022_2505_MOESM10_ESM.tif]
