# Supplementary material for: Exosomal circZNF451 restrains anti-PD1 treatment in lung adenocarcinoma via polarizing macrophages by complexing with TRIM56 and FXR1
Source: J Exp Clin Cancer Res. 2022 Oct 8;41:295. doi: 10.1186/s13046-022-02505-z (PMC9547453; doi:10.1186/s13046-022-02505-z)
Supplement: Supplementary file 11 — Additional file 11. Supplementary methods. [file 13046_2022_2505_MOESM11_ESM.docx]

**Supplementary Methods**

**Western blot**

The cells lysed by the RIPA lysis buffer and the protease inhibitor (Yeasen, China) were mixed with the loading buffer. After the separation by sodium dodecyl sulfate-polyacrylamide gel electrophoresis, the protein was transferred onto the PVDF membranes and blocked by the rapid blocking buffer (Yeasen, China) for 15min. Then the membranes were firstly incubated with the primary antibodies overnight and then incubated with the secondary antibodies for 1h. The bands were detected with a chemiluminescence imaging system (Tanon, Shanghai, China).

**qRT-PCR**

The total RNA in the cells was extracted with the TRIeasyTM Total RNA Extraction Reagent (Yeasen, China). Then, the it was reversed into the cDNA with the Hifair® Ⅲ 1st Strand cDNA Synthesis SuperMix according to the manufacturer’s protocol. Next, the reaction system was created with the SYBR Green Real-Time PCR Master Mix (Yeasen, China) and detected on an Applied Biosystem. The separation of RNA in nucleus and cytoplasm was performed according to the manufacturer’s protocol (Norgen biotek, Canada). The mRNA of GAPDH was applied as the internal parameter.

**IHC**

After the dewaxing and rehydration, the tissues were blocked by 3% H_2_O_2_ for 30min and recovered by the citric acid retrievals at 100°C for 20min. Then, the tissues were blocked by bovine serum albumin and incubated with the primary antibodies overnight and further incubated with the secondary antibody for 1h. Next the slides were stained by DAB. Finally, the tissues were stained by hematoxylin. The primary antibodies for CD8, CD163 and CD86 were recorded in supplementary table 2. To count the number of CD8^+^T cells and CD163^+^/CD86^+^macrophages, we selected four representative high-power (200×) fields and the average number of positive cells were regarded the final number.

**Subcutaneous xenograft model**

A total of 5×10^6^ LLC-circZNF451 cells were suspended in 100μl DMEM and subcutaneously implanted into the right flank of C57BL/6J and the transgenic mice model with the conditional knockout of ELF4 in macrophage. The tumor size was measured every 3 days until fortnight. The tumor size equals 1/2length× width^2^. The PD1 blockade was administered 6 days later and was i.p given every three days. The dose was 1mg/kg for one mouse.
