# Supplementary material for: Exosomal circZNF451 restrains anti-PD1 treatment in lung adenocarcinoma via polarizing macrophages by complexing with TRIM56 and FXR1
Source: J Exp Clin Cancer Res. 2022 Oct 8;41:295. doi: 10.1186/s13046-022-02505-z (PMC9547453; doi:10.1186/s13046-022-02505-z)
Supplement: Supplementary file 12 — Additional file 12: Supplementary Table 1. The clinical information of six LUAD patients accepted the PD1 blockade and underwent circRNA-seq of the peripheral blood. Supplementary Table 2. The clinical characteristics of 113 LUAD patients with different expression of circZNF451. Supplementary Table 3. Target sequences of shRNA and siRNA used in this study. Supplementary Table 4. The antibodies, ELISA kits and drugs used in this study. Supplementary Table 5. The primers and probes used in this study. Supplementary Table 6. The validation set of 10 LUAD patients accepting the PD1 blockade. [file 13046_2022_2505_MOESM12_ESM.docx]

Supplementary table 1: The clinical information of six LUAD patients accepted the PD1 blockade and underwent circRNA-seq of the peripheral blood.

| Patient | Sex | Age | Pathology | Smoking | T (cm) | N | M | Response |
| --- | --- | --- | --- | --- | --- | --- | --- | --- |
| 1 | Female | 33 | LUAD | No | 5.2 | N2 | lung， pleura | PD |
| 2 | Female | 46 | LUAD | No | 6.5 | N2 | adenral gland, lung | PD |
| 3 | Male | 59 | LUAD | Yes | 4.1 | N1 | bone | PD |
| 4 | Male | 49 | LUAD | No | 4.9 | N2 | lung， pleura | PR |
| 5 | Male | 61 | LUAD | No | 3.8 | N2 | brain | PR |
| 6 | Female | 52 | LUAD | No | 5.3 | N1 | brain, lung | PR |

Note: LUAD, lung adenocarcinoma; PD, progressive disease; PR, partial remission

Supplementary table 2: The clinical characteristics of 113 LUAD patients with different expression of circZNF451

| variables |  | circZNF451 expression | |  |
| --- | --- | --- | --- | --- |
|  |  | low | high | P |
| Sex |  |  |  | 0.108 |
|  | Male | 22 | 31 |  |
|  | Female | 34 | 26 |  |
| Age (year) |  |  |  | 0.289 |
|  | ≤ 60 | 35 | 30 |  |
|  | > 60 | 21 | 27 |  |
| Smoking status |  |  |  | 0.636 |
|  | Smokers | 26 | 29 |  |
|  | Nonsmokers | 30 | 28 |  |
| Tumor size (cm) |  |  |  | 0.024 |
|  | ≤ 3 | 41 | 30 |  |
|  | > 3 | 15 | 27 |  |
| Differentiation |  |  |  | 0.734 |
|  | Well and moderate | 17 | 19 |  |
|  | Poor | 39 | 38 |  |
| Lymph node metastasis |  |  |  | 0.029 |
|  | Yes | 19 | 31 |  |
|  | No | 37 | 26 |  |
| TNM |  |  |  | 0.027 |
|  | I-II | 45 | 35 |  |
|  | III-IV | 11 | 22 |  |

Supplementary table 3: Target sequences of shRNA and siRNA used in this study

| shRNA and siRNA | target sequence (5'→3') |
| --- | --- |
| shcircZNF451#1 | ACAGAAGGAGGAAACACCAAT |
| shcircZNF451#3 | TTATATCCACAGAAGGAGGAA |
| siFXR1#3 | CGCCAGGTTCCATTTAATGAA |
| siTRIM56#1 | GCAGCAGAATAGTGTGGTAAT |
| siTRIM56#2 | CCTGGACTGTGCCGATGACTT |
| siELF4#1 | AGAGGCTGGTGTACCAGTTTA |
| siELF4#2 | GATCTTCAGTACCTCCGAAAT |
| siIRF4#1 | CCAGCAGGTTCACAACTACAT |

Supplementary table 4: The antibodies, ELISA kits and drugs used in this study.

| Antibodies，ELISA kits and drugs | Company | Cat No |
| --- | --- | --- |
| Anti-FXR1 | Abcam | ab245624/ab129089 |
| Anti-TRIM56 | Abcam | ab154862 |
| Anti-TRIM25 | Abcam | ab167154 |
| Anti-ELF4 | Santa Cruz | sc-390689X |
| Anti-IRF4 | CST | 62834 |
| Anti-Ubiquitin | Abcam | ab134953 |
| Anti-Flag | Abcam | ab205606 |
| Anti-TSG101 | Abcam | ab125011 |
| Anti-CD63 | Abcam | ab134045 |
| Anti-CD8 | CST | 85336 |
| Anti-CD163 | CST | 93498 |
| Anti-CD86 | CST | 76755 |
| Anti-GAPDH | Abcam | ab8245 |
| 7-AAD Viability Staining Solution | Biolegend | 420404 |
| APC/Cyanine7 anti-mouse/human CD11b | Biolegend | 101226 |
| FITC anti-mouse CD3 | Biolegend | 100203 |
| Alexa Fluor® 700 anti-mouse F4/80 | Biolegend | 123129 |
| PE/Cyanine7 anti-mouse CD11c | Biolegend | 117318 |
| Brilliant Violet 510™ anti-mouse CD4 | Biolegend | 100559 |
| Brilliant Violet 605™ anti-mouse CD8a | Biolegend | 100744 |
| PE anti-mouse FOXP3 | Biolegend | 126403 |
| PerCP anti-mouse CD45 | Biolegend | 103130 |
| Anti-Mouse NK1.1 Alexa Fluor®488 | Cedarlane | CL8994AF4 |
| Anti-Mouse CD32/CD16, Purified (rat IgG2a) | Biolegend | MCD03216-M |
| Brilliant Violet 421™ anti-mouse CD163 | Biolegend | 155309 |
| PE anti-mouse CD86 | Biolegend | 105007 |
| PE/Cyanine7 anti-mouse IFN-γ | Biolegend | 505826 |
| Alexa Fluor® 647 anti-human/mouse Granzyme B | Biolegend | 515405 |
| PE anti-human CD279 (PD-1) | Biolegend | 329905 |
| Brilliant Violet 421™ anti-human CD366 (Tim-3) | Biolegend | 345007 |
| APC anti-mouse TIGIT (Vstm3) | Biolegend | 142105 |
| Human CXCL1/GRO alpha DuoSet ELISA | R&D system | DY275 |
| Human GM-CSF DuoSet ELISA | R&D system | DY215 |
| Human IL-1 beta/IL-1F2 QuicKit ELISA | R&D system | QK201 |
| Human IL-1ra/IL-1F3 DuoSet ELISA | R&D system | DY280 |
| Human IL-10 DuoSet ELISA | R&D system | DY217B |
| MG-132 | Yeasen | 52801ES08 |
| Murine Anti-mPD-1 mAb | InvivoGen | mpd1-mab15-10 |
| GW4869  CFSE Cell Division Tracker Kit | Yeasen  Biolegend | 52321ES05  423801 |

Supplementary table 5: The primers and probes used in this study

| Gene |  | Primer or sequence （5'→3'） |
| --- | --- | --- |
| circZNF451 | Forward primer | TCTGATACCACCAAAGAATGTGAC |
|  | Reverse primer | TCTTACAGTTGAGCGGAGGC |
| has-circ-0008790 | Forward primer | GCCACACTGGAGAGATTAGAAGG |
|  | Reverse primer | CCTCTTCTTCATCAGCAACAGGT |
| has-circ-0079395 | Forward primer | TATGGAGGTTTCTCACACTGGT |
|  | Reverse primer | TCCTTGCTCCAACTTGCTGA |
| has-circ-0004784 | Forward primer | AGACAAAGTCAACTCAACTGTGG |
|  | Reverse primer | GGAACCTTCGCCTGCATCTA |
| ZNF451 | Forward primer | AATGCCAGGACTCAAAACAGGCA |
|  | Reverse primer | GAGAGTGATCGAACCTTTTCAAA |
| FXR1 | Forward primer | CCCTAATTACACCTCCGGTTATG |
|  | Reverse primer | TCTCCTGCCAATGACCAATC |
| Arg1 | Forward primer | CTTAAAGAACAAGAGTGTGATGTGA |
|  | Reverse primer | ATTGCCAAACTGTGGTCTCC |
| IL-10 | Forward primer | AGACAGACTTGCAAAAGAAGGC |
|  | Reverse primer | TCGAAGCATGTTAGGCAGGTT |
| ELF4 | Forward primer | GCTCGTTTCGGTGCATGTCT |
|  | Reverse primer | AAATACCAGGTGAGACGGCG |
| IRF4 | Forward primer | TCCGACAGTGGTTGATCGAC |
|  | Reverse primer | CCTCACGATTGTAGTCCTGCTT |
| GAPDH | Forward primer | TCGGAGTCAACGGATTTGGT |
|  | Reverse primer | TTCCCGTTCTCAGCCTTGAC |
| circZNF451 | probe | ACCTTGGTGTTTCCTCCTTCTGTGGATAT |

Supplementary table 6: The validation set of 10 LUAD patients accepting the PD1 blockade

| Patient | Sex | Age | Pathology | Smoking | T (cm) | N | M | Response |
| --- | --- | --- | --- | --- | --- | --- | --- | --- |
| 1 | Male | 68 | LUAD | Yes | 5.8 | N1 | lung, pleura | PR |
| 2 | Female | 53 | LUAD | No | 6.2 | N2 | bone | PR |
| 3 | Female | 39 | LUAD | Yes | 4.5 | N2 | lung, pleura | PR |
| 4 | Male | 57 | LUAD | No | 6.5 | N2 | bone, adrenal gland | PR |
| 5 | Female | 56 | LUAD | No | 4.1 | N1 | pleura, bone | PD |
| 6 | Male | 48 | LUAD | No | 3.9 | N3 | brain | PD |
| 7 | Female | 54 | LUAD | No | 4.4 | N2 | lung, bone | PD |
| 8 | Male | 48 | LUAD | Yes | 6.1 | N2 | lung, pleura | PD |
| 9 | Male | 59 | LUAD | No | 2.8 | N1 | bone | PD |
| 10 | Female | 63 | LUAD | No | 3.9 | N2 | pleura | PD |

Note: LUAD, lung adenocarcinoma; PD, progressive disease; PR, partial remission
